# The role of maternal infections in neurodevelopmental psychiatric disorders: focus on the P2X7/NLRP3/IL-1β signalling pathway

**DOI:** 10.1186/s12974-025-03509-0

**Published:** 2025-07-26

**Authors:** Dorottya Szabó, Lilla Otrokocsi, Beáta Sperlágh

**Affiliations:** 1 Laboratory of Molecular Pharmacology, HUN-REN Institute of Experimental Medicine, Szigony u. 43, Transpose, Hungary; 2 János Szentágothai Doctoral School, Semmelweis University, Üllői út 26, Budapest, H-1085 Hungary

**Keywords:** Neurodevelopmental disorders, Maternal immune activation, Prenatal infection, P2X7, NLRP3, Interleukin-1β, Neurodevelopment

## Abstract

Immune activation in the prenatal and early postnatal periods is increasingly implicated in the aetiology of neurodevelopmental disorders, such as autism spectrum disorder and schizophrenia, by disrupting critical neurodevelopmental processes. The impact of immune activation on brain development can be influenced by the type, timing, location, and severity of the infection. Viral, bacterial, and parasitic infections, as well as maternal autoimmune diseases, can lead to the activation of the purinergic P2X7 receptors, thereby contributing to neuroinflammation. Upon activation, P2X7 induces the assembly of the NLRP3 inflammasome, leading to the release of the pro-inflammatory cytokine IL-1β. Besides activation of additional inflammatory mediators, excessive IL-1β during critical periods of brain development can disrupt neuronal migration, synapse formation, dendritic morphology and blood-brain barrier integrity, contributing to a range of neurodevelopmental abnormalities. Animal studies have shown that inhibiting the components of the P2X7/NLRP3/IL-1β pathway can mitigate these adverse effects. This review examines the role of the P2X7/NLRP3/IL-1β pathway in mediating the effects of infection and neuronal inflammation on brain development. We discuss the therapeutic potential of targeting this pathway with a balanced approach that reduces long-term neuronal inflammation while preserving essential immune functions.

## Highlights

Maternal immune activation is increasingly linked to ASD and schizophrenia.

Maternal infection and autoimmunity: persistent in-utero inflammation via P2X7/NLRP3/IL-1β.

Elevated IL-1β disrupts neuronal migration and synapse formation.

Prenatal inflammation is linked to lifelong learning and social challenges.

P2X7/NLRP3/IL-1β pathway is a potential therapeutic target for future research.

## Introduction

The underlying etiological and pathological features of neurodevelopmental disorders such as autism spectrum disorder and schizophrenia remain elusive and are the subject of active research. The aetiology of both disorders is considered multicausal, involving genetic and environmental factors. Recently, the focus of research has shifted from genetic to epigenetic mechanisms of environmental effects, highlighting the increasingly prominent role of the latter. Immune activation during pregnancy has been suggested as one of the environmental factors associated with neurodevelopmental deficits.

Maternal immune activation (MIA) during pregnancy triggered by infections, autoimmune conditions, allergies, or immunological deficiencies can cause persistent in-utero inflammation. Elevated extracellular ATP, an early danger signal, activates immune and endothelial cells [1], influencing neuronal activity via purinergic receptors and contributing to neurodegenerative and inflammatory changes in the central nervous system [2].

Purinergic receptors are plasma membrane molecules found abundantly in all mammalian tissues, predominantly in neurons and glial cells, muscle cells, epithelial cells, endothelial cells, endocrine cells, bone cells and all cells of innate and adaptive immunity. P2X receptors play a central role in maintaining inflammation, particularly the P2X7 receptor, by inflammasome assembly through NLRP3 activation and release of inflammatory cytokines, such as IL-1β [3]. Therefore, the P2X7-NLRP3 route is crucial for converting the body’s innate immune response into inflammation [4, 5]. IL-1β is an important mediator of the inflammatory response and, besides fever induction, is involved in a variety of cellular activities, including cell proliferation, differentiation, and apoptosis, as well as in the induction of behavioural changes [6]. It is also able to recruit other proinflammatory cytokines [7] and is involved in the modulation of autoimmune inflammation [8]. The activated cytokines and inflammatory mediators can alter fetal brain development, impacting neuro- and gliogenesis, neuronal migration, synaptogenesis, and the elimination of synapses during development, resulting in the formation of abnormal cortical and cerebellar neural networks [9, 10].

In this review, we focus specifically on how maternal immune activation (MIA) — induced by infections, autoimmune diseases, or other immune challenges during pregnancy — affects fetal brain development via the P2X7/NLRP3/IL-1β signalling axis. While the involvement of P2X7 in neurodevelopment and inflammation has been broadly described, our aim was to integrate recent evidence on how this pathway is activated in the maternal-fetal context and how it contributes to long-term neurodevelopmental disorders. This targeted approach provides a novel framework to understand the immune–neurodevelopmental interface.

## Activation of the P2X7/NLRP3/IL-1β signalling pathway

### Role of P2X7 in inflammatory ATP signalling

The P2X7/NLRP3/IL-1β pathway is essential for maintaining and amplifying inflammation and can lead to long-term inflammatory effects through an ATP feedback loop. ATP is stored in synaptic vesicles and astrocyte vesicles and can be released from nerve terminals, dendrites and axons [11], astrocytes [12] and microglia [13] through multiple mechanisms [14]. During physiological neuronal activation, ATP is released, which acts as a homeostatic messenger to mediate cell-cell communication, with P2X7 receptors on surrounding cells being activated by ATP to influence cellular signaling, contributing to homeostasis [15]. In conditions such as inflammation and hypoxia, extracellular ATP levels can markedly rise due to active release and passive leakage from damaged or dying cells, along with decreased levels of ectonucleotidases [16]. Several ion channels like connexin hemichannel, pannexin 1 (Panx1), volume-regulated anion channels (VRACs), maxi-anion channels, and calcium homeostasis modulator 1 (CALHM1) might be involved in ATP-release [17]. The increase of extracellular ATP concentration activates cell surface P2X and P2Y receptors. Purinergic P2X receptors are homo- (P2X7) or heteromeric non-selective ligand-gated membrane-bound cation channels, formed by three subunits of the P2X1-7 subtypes. They are widely expressed in the nervous system by central and peripheral neurons and glial cells [18]. P2X receptors open upon ATP binding, leading to cation influx, membrane depolarisation or calcium-dependent enzymatic signal transduction [19]. P2X7, in particular, has a lower affinity for ATP (0.1–1 mM) compared to other P2X, suggesting that their activation mainly occurs under pathological conditions associated with enhanced extracellular ATP levels [20, 21]. This occurs, for example, in the case of an inflammatory environment or the destruction of surrounding cells due to trauma, hypoxia or other adverse events. Another characteristic of P2X7 is that long-term, high extracellular concentration of ATP leads to prolonged P2X7 activation resulting in rapid Ca^2+^ and Na^+^ influx with K^+^ efflux, which can be followed by the formation of a large non-selective transmembrane pore on the cell surface for molecules up to ~ 900 Da that induces cell lysis/necrosis or apoptosis [22].

### Interplay of P2X7 and pannexin-1 during inflammation

The increase of intracellular calcium induced by P2X7 can also activate pannexin-1 in the early stages of innate immunity, leading to membrane permeabilisation and the release of macromolecules [23]. Pannexins are hemichannels present in plasma membrane, endoplasmic reticulum and Golgi membranes and can form nonjunctional transmembrane channels for transporting molecules of less than 1000 Da. Although it is generally accepted that pannexin-1 has cytotoxic effects [24], pannexin-1 may also have a protective function against cell death [23, 25]. Some studies describe that Panx1 and P2X7R may have regulatory effects on each other based on their co-localization, physical interactions and involvement in convergent activities [23, 26]. However, the ATP-conductance and -release properties of pannexin-1 are not entirely determined [27], and the mechanism of the pannexin-1 opening is not fully understood [28]. Overall the issue is highly controversial, but increasing the activation time, either by dilation of the ionic pore of the P2X7 receptor or by opening large-conductance accessory proteins, like hemichannels [29], enables the uptake of large cations and the further release of ATP from the cells and hence contributes to further P2X7 and other purinergic activation. It is likely that both P2X7 and pannexin-1 play a prominent role in the non-synaptic communication of the CNS, especially during inflammatory conditions.

### TLR-induced priming and P2X7-mediated activation of the NLRP3 inflammasome

**Fig. 1:**
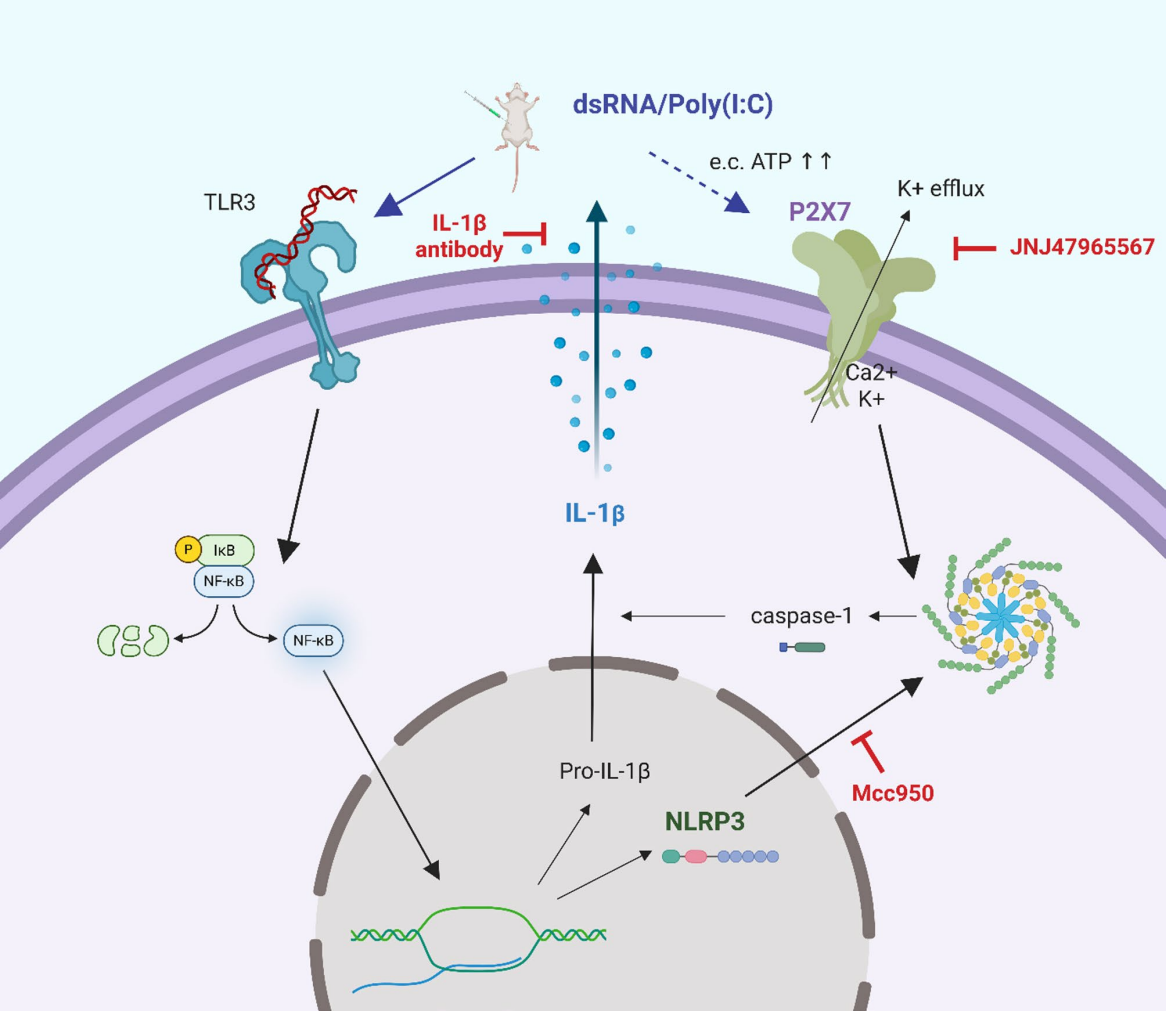
Activation of the P2X7/NLRP3/IL-1β signalling pathway in MIA animal models. TLR3 recognises double-stranded RNA, such as poly(I:C), leading to NF-κB-dependent upregulation of inflammatory cytokine precursors and NLRP3 components. Increased extracellular ATP then activates P2X7, resulting in the formation of NLRP3 inflammasome. This complex activates caspase-1 via dimerisation and self-cleavage. Active caspase-1 processes pro-IL-1β into its biologically active form by proteolysis, ready to be released into the extracellular space

Infections trigger a response in the body, where external danger stimuli like viral RNA or bacterial lipopolysaccharide sequence interact with Toll-like receptors (TLRs) [30]. TLRs are innate immune receptors expressed in immune, glial and neuronal cells of the central and peripheral nervous system [31]. TLRs play a role in both infectious and non-infectious responses in the CNS and can modulate glial and neuronal function as well as innate immunity and neuroinflammation in both physiological and pathophysiological conditions such as infections, CNS autoimmunity, neurodegeneration and tissue injury [31, 32]. Specific TLRs recognise distinct PAMPs: TLR3 recognises double-stranded RNA, TLR4 recognises bacterial lipopolysaccharide (LPS), TLR5 interacts with bacterial flagellin [33], TLR7 and TLR8 recognise intracellular single-stranded RNA such as viral ligands, nucleosides, and oligoribonucleotides [34, 35], and TLR9 recognises bacterial or viral CpG DNA motifs [36]. Ligand binding of TLR receptors leads to the transcriptional upregulation of inflammatory cytokine precursors (Fig. 1), such as pro-IL-1β, pro-IL-18 and NLRP3, in a NF-κB dependent manner [37]. Some studies propose that post-translational modifications of NLRP3, like phosphorylation and ubiquitination, may also contribute to this priming phase [38, 39]. The primed cell in this phase needs to be exposed to a new PAMP or DAMP (pathogen- or damage-associated molecular patterns) to induce the processing and secretion of an active IL-1β molecule. As part of a second, activation step induced by different DAMPs, the increase in extracellular ATP then activates P2X7, resulting in the formation of NLRP3 inflammasome [40]. Although the exact mechanism of NLRP3 activation is not yet fully understood, K^+^ efflux, Ca^2+^ signalling, Na^+^ influx, and chloride efflux have all been identified as critical events in the process, of which cellular K^+^ efflux is the most important and well-identified step in NLRP3 inflammasome activation [37, 38, 41, 42]. While intracellular K^+^ depletion is important, some studies suggest that reactive oxygen species production can activate the NLRP3 inflammasome independently of K^+^ efflux [43]. P2X7 may contribute to NLRP3 activation through a combination of mechanisms of a decrease in intracellular K^+^, an increase in intracellular Ca^2+^, induction of ROS production, depolarisation of mitochondria and destabilisation of lysosomes [4, 38, 41, 42, 44]. The NLRP3 inflammasome consists of the NLRP3 sensor molecule, the ASC adaptor protein and pro-caspase-1. Upon activation, NLRP3 interacts with ASC, which then recruits pro-caspase-1, forming the NLRP3 inflammasome. This complex activates caspase-1 through dimerisation and self-cleavage [45]. Active caspase-1 is a cysteine protease, which processes pro-IL-1β into its biologically active form by proteolysis, ready to be released into the extracellular space [46]. Interestingly, a recent study has shown that P2X7 is capable of inducing an NLRP3-independent increase in IL-1β release, as ATP-induced P2X7-mediated IL-1β release was measured in primed macrophages in the presence of pharmacological and genetic NLRP3 depletion [47]. Further research is needed to evaluate this activation mechanism.

### IL-1β release and signalling in the CNS

There are several possible mechanisms through which IL-1β could be released from the cells, such as regulated secretion of lysosomes [48], the shedding of microvesicles from the plasma membrane [49], release from exosomes together with entrapped caspase-1 and other inflammasome components [50], its direct release through a hyperpermeable plasma membrane during cell death [51] or pyroptosis [52]. IL-1β is now known to be produced directly by cells of the CNS, including microglial cells, astrocytes, oligodendrocytes, neurons, and all of these cell types are also capable of responding to the cytokine. IL-1β has a pleiotropic effect in the CNS. It acts as a neuromodulator in the healthy brain and is involved in synaptic plasticity and the pathogenic process of many CNS diseases [10]. It can stimulate IL-6 production in astrocytes and neurons and inducible nitric oxide synthase (iNOS) activity in astrocytes [53, 54] contributing to inflammatory responses. IL-1β exerts its well-known pro-inflammatory effects by binding to the IL-1R1 receptor, whereas it can also bind to IL-1R2 to exert anti-inflammatory effects. IL-1R3 acts as a co-receptor, forming a trimeric signalling complex with IL-1β and IL-1R1. Under physiological conditions, IL-1R1 and IL-1R3 are present on the cell membrane, and can also be cleaved by matrix metalloproteases to a soluble, circulating form. Both the membrane-bound and the soluble forms are biologically active [55]. IL-1β binding to IL-1R1 induces a conformational change allowing IL-1R3 binding [55]. The resulting complex triggers TIR domain dimerisation, recruiting MyD88 and initiating a signalling cascade involving IRAKs and TRAF6. Subsequently, multiple intracellular phosphorylation and ubiquitination processes lead to the activation of mitogen-activated protein kinase (MAPK) p38, the c-Jun N-terminal kinase (JNK) and nuclear factor kappa B (NF-κB) [56]. These changes result in the upregulation of mRNA transcription for inflammation-associated genes encoding IL-6, IL-8, iNOS, monocyte chemoattractant protein-1 (MCP-1), cyclooxygenase-2, IκBα, IL-1α, IL-1β and MAPK phosphatase 1 [57]. The endogenous interleukin-1 receptor antagonist (IL-1Ra) can counteract IL-1R1 by inhibiting the association of IL-1R1 with IL-1R3, thus competitively blocking IL-1β signalling [56]. IL-1R2, a decoy receptor, sequesters IL-1β, existing in both membrane-bound and soluble forms. Soluble IL-1R2 neutralises IL-1β, especially when complexed with soluble IL-1R3. IL-1R2, existing in both membrane-bound and soluble forms, is a decoy receptor for IL-1β, lacks a cytoplasmic domain (TIR), and does not signal but sequesters IL-1β. Although soluble IL-1R2 binds IL-1β in the extracellular space, neutralisation of IL-1β activity is greatly enhanced by forming a complex with soluble IL-1R3 [55].

### Cell-type specific signalling and the complexities of IL-1β action in the CNS

In the nervous system, IL-1R1 expression is driven by multiple cell type-specific promoters, allowing cell type-specific control of IL-1R1 expression [58]. Thus, diverse IL-1β effects in the brain likely result from distinct cell-type-specific IL-1R1 signalling pathways (e.g., p38 MAPK, NF-κB), which can be activated individually or in combination depending on the tissue or cell type [56, 59]. However, investigating cell-type-specific IL-1β expression and signalling remains challenging, and some studies show contradictory data [58]. Similarly, the precise CNS locations where peripheral IL-1β exerts its effects remain uncertain. It has long been suggested that IL-1β can cross the blood-brain barrier (BBB) [60]. During neuroinflammation, IL-1β is known to stimulate the expression of adhesion molecules on endothelial cells that facilitate the attachment and migration of immune cells across the BBB [61]. However, the role of endothelial IL-1R1 at the BBB in mediating its effects and the mechanisms of crossing remains uncertain [58].

## The P2X7/NLRP3/IL-1β signalling pathway in neuronal development

### Function and role of P2X7 receptors in neuronal development

Prenatal development: During embryonal neurogenesis, P2X7 subunit expression starts at E14-15 in rat brain [62–64] and continues through postnatal development (P16) [65]. P2X7 plays a conducting role in the alteration of cellular functions according to the current developmental age until adult neurogenesis. P2X7 regulates crucial aspects of neuronal cell biology, including axonal elongation, path-finding, synapse formation, and neuroprotection, and modulates neuroinflammation [66, 67]. Similarly to adult neurogenesis, P2X7 controls the proliferation and alters cell cycle progression in embryonic development processes, but does not cause cell death [64]. The upregulation of P2X7 receptor expression and activity in embryonic cells has been determined to contribute to maintaining embryonic stem cell proliferation, while upon induction to neural differentiation, P2X7 receptor expression and activity need to be suppressed as the onset of neuroectodermal differentiation and neuronal fate determination depends on the suppression of the receptor-activity [68]. An immunofluorescence staining experiment proved that P27 was expressed in both the soma and neuronal processes of the cells in 3-day-old neonatal rat brain samples. This study also showed that P2X7 is widely expressed in all layers of the prefrontal cortex in neonatal rats [69]. Sebastián-Serrano et al. performed in-utero cortical electroporation to define the involvement of P2X7 in the axonal elongation deficits associated with neurological disorders [67]. According to an in vitro mouse study, P2X7 also regulates dendritic outgrowth and branching in the early stages of development [70]. Prenatal and early life factors that disrupt physiological brain development contribute to behavioural and neurochemical changes in adulthood by altering the purinergic system [71]. *Adult neurogenesis*: Beyond early development, P2X7 receptors also play a crucial role in adult neurogenesis and neuroregeneration. In the postnatal brain, P2X7 has a leading role in the maintenance of neuronal physiology, including axonal elongation, branching, and neurotransmitter release [72]. Neural progenitor cells can differentiate into neurons, astrocytes, and oligodendrocytes. The expression of P2X7 receptors on progenitor cells has been demonstrated for a long while [73]. P2X7 receptors play a role in the continuous sustain and quantitative regulation of the neural stem cell pool [74]. In the adult mammalian hippocampus, neurogenesis is a persistent and essential feature in which P2X7 has diverse regulatory functions, notably inducing cell death via transmembrane pore formation [75], modulating glutamate release and neurotrophic factor signaling, such as BDNF [76]. These receptors can also act as scavenger receptors in microglia. Furthermore, cellular debris may be cleared by neural progenitors and neuroblasts via P2X7-mediated phagocytosis as well. The receptor also has potential roles in the regulation of proliferation, differentiation, and axonal extension (Leeson et al., 2019). The above-mentioned pore formation, induced by an increase in extracellular ATP concentration, results in cell lysis/necrosis or apoptosis, depending on the nature of the stimulus, the duration of the stimulus and the cell type [22]. Thus, during CNS-related adverse events involving high inflammation and ATP release, P2X7 receptors can delay regeneration and cell renewal.

### Function and role of NLRP3 in neuronal development

The role of NLRP3 has been widely described in CNS disorders, but very little data is available on its role in normal brain development. As a result of rapid neuronal cell proliferation, many neurons die during neurodevelopment. Uniquely, Lammert et al. demonstrated in mouse brains that the innate immune system plays a crucial role in the clearance of damaged cells, releasing ATP, damaged mitochondria and ROS to the extracellular space in the normally developing nervous system by pyroptosis [77], which is a regulated form of lytic cell death necessary for proper CNS development and function [78]. They observed high levels of ASC staining throughout the brain at postnatal day 5, confirming inflammasome assembly at this time point. However, they found that while loss of AIM2 inflammasome (activated by cytosolic DNA) and caspase-1 in mouse studies resulted in anxiety-like behaviours in the elevated plus maze and open field, loss of NLRP3 did not affect animal behaviour [77]. In agreement, the transcripts of ASC and caspase-1, which are both downstream mediators of NLRP3, appear to be detectable in early human fetal neurons, astrocytes, and BMCs (Brain Macrophage-like Cells) under normal neurodevelopmental conditions, while NLRP3 transcript seems to be inducible only when exposed to a priming stimulus [79]. Further studies are needed to clarify or exclude the role of NLRP3 in physiological brain development.

### Function and role of IL-1β in normal neuronal development

IL-1β is highly expressed in the brain during prenatal and postnatal development and declines to low levels in adults, suggesting an essential role in brain development [80]. In human embryonic forebrain cells, constitutive IL-1β expression was detected from week 5, increased by approximately 50% at week 7, and was highest at week 10 [81]. Similarly, IL-1 has been detected in rat embryos from E14 until adulthood, with the highest expression at E18 [82]. According to in vitro animal studies, IL-1β plays an important role in the migration of cortical neurons in a dose-dependent manner [80] and of astrocyte progenitor cells by modulating calcium signalling [83] during early stages of CNS development. IL-1β participates in guiding leading process endings, the so-called growth cone, of migrating neurons, contributing to the control of migration direction [80]. Furthermore, in vitro studies also show that IL-1β is involved in the regulation of neurite outgrowth, which is an essential process during early neuronal development. In more detail, IL-1β acts synergistically with neurotrophin-3 and both stimulate neurite density and length potentially via the Wnt5a/RhoA/JNK pathway [84]. The cortical neurons also express IL-1R1 in vitro and in vivo [80]. Both the dysregulation of cell migration and neurite outgrowth can lead to autism spectrum disorder (ASD) and other neurodevelopmental disorders [85].

## Pathogenic factors that can affect fetal brain development and their relations to the P2X7R/NLRP3/IL-1β pathway

### Maternal infections during pregnancy

While over half of pregnant women experience at least one infection [86], in most cases no brain disorders develop in the fetus. However, it has been increasingly recognised that various infections during pregnancy poses a higher risk of later onset of various neurodevelopmental disorders during childhood and adolescence [87] a line of research supported by a large number of clinical studies. Viral infection’s impact on neurodevelopmental risk depends on various factors, including type, timing, severity, and location. A link exists between epidemics/pandemics and increased schizophrenia incidence [88]. Nevertheless, many unknown factors still need to be explored to understand the connections between risk factors and timing.

**Fig. 2:**
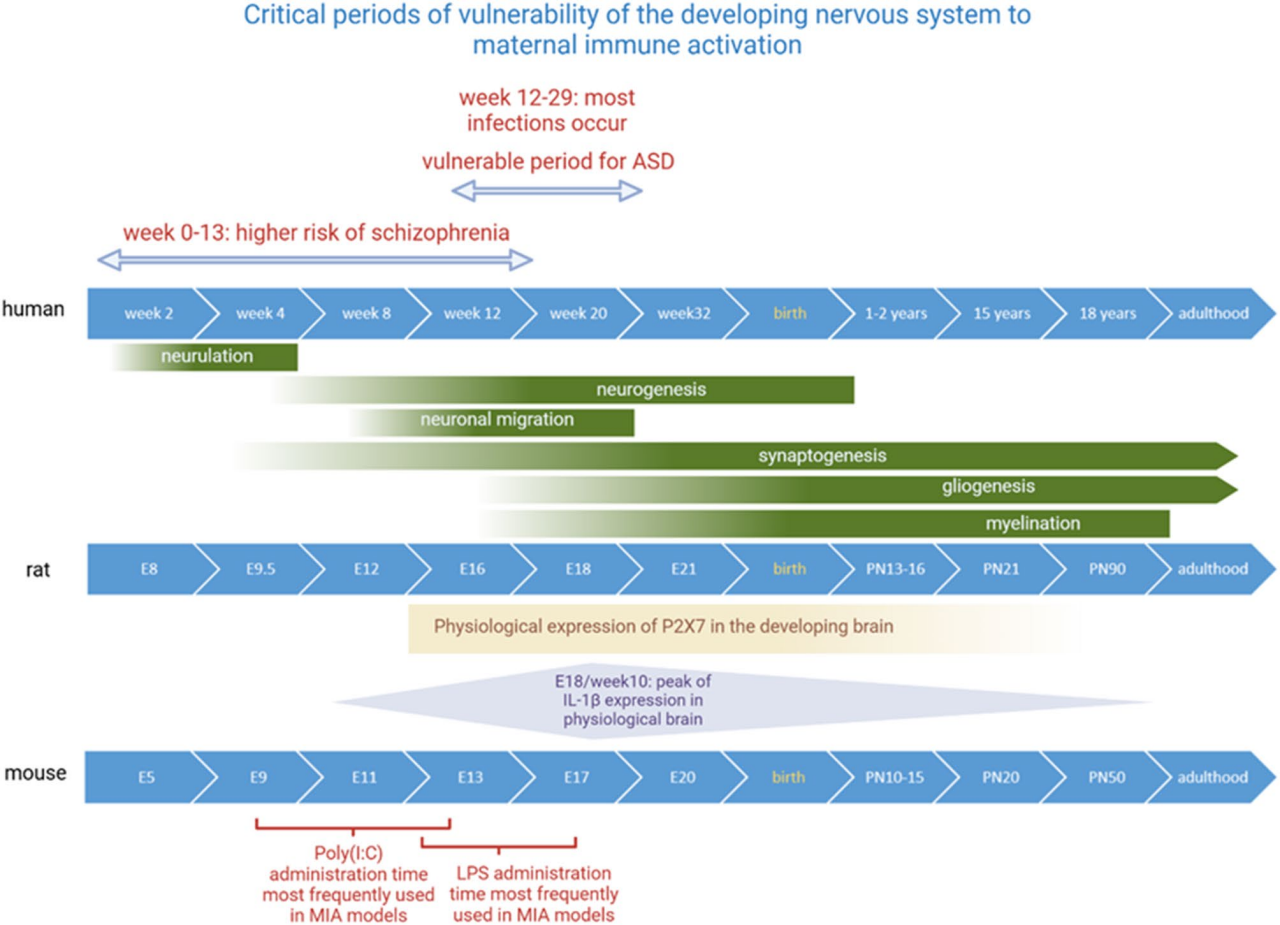
Critical periods of vulnerability of the developing nervous system to maternal immune activation. A comparative overview of the timing of neurodevelopmental processes and vulnerability tomaternal immune activation across human, rat, and mouse models. The developing nervous system, including that of the human fetus, exhibits varying vulnerability to maternal immune activation throughout gestation. First-trimester infections are associated with a higher risk of schizophrenia, while second-trimester infections are linked to an increased risk of ASD. Third trimester infections show a slight association with increased ASD risk. The timelines of key developmental processes (e.g., neurogenesis, gliogenesis, synaptogenesis) are aligned across species to highlight translationally relevant windows. The expression of P2X7, starting at E14-15in the rat brain and continuing through postnatal development, and the high prenatal andpostnatal expression of IL-1β (detectable from E14, peaking at E18, and declining to low adult levels), suggest their crucial roles in brain development during these vulnerable periods. Maternal immune activation during these stages could disrupt these critical processes, potentially contributing to neurodevelopmental disorders.

In the first trimester of pregnancy (0–13 weeks in humans), the ectoderm folds and fuses to form the neural tube during the second and third weeks (neurulation) [89, 90]. In the fourth week, the rostral part of the neural tube forms three vesicles that will give rise to the forebrain, midbrain and hindbrain. By weeks 5–6, neuronal precursors undergo rapid proliferation, which is genetically determined and epigenetically controlled by the environment [89]. By week 8, neuroblasts differentiate into specific neuronal cell types or macroglia (neurogenesis). These neurons migrate to the cortical layer and eventually form synapses during the late first to second trimester (12–29 weeks) [89]. During the third trimester, glial precursors form into neuronal axons and glia such as astrocytes and oligodendrocytes (gliogenesis) [91]. Brain morphology and plasticity continue to develop after birth [89]. While the sequence of these developmental processes is conserved across mammals, analogous events in rodents occur on a shorter gestational timeline (Fig. 2). Most infections during human pregnancy occur in the second trimester [86], which can be linked to qualitative immunological changes or a more active lifestyle in the second trimester. The first and second trimesters are most vulnerable to prenatal infections causing long-term neuronal changes [92]. Primarily, the first trimester has been associated with a higher risk of schizophrenia [93–95], while the risk of developing ASD is more linked to maternal infection during the second trimester, when the fetal brain is undergoing significant growth and differentiation, but not in the first [96–98]. Third-trimester infection has been slightly associated with an increased risk of ASD [96]. Recent studies have shown that third-trimester infections increase the risk of cognitive impairments, especially performance IQ [86, 99]. Associations between bacterial infections in the third trimester and cognitive impairments in early childhood seem to be strongest among males and for more severe infections or multi-systemic infections [99]. One study suggests that maternal infection in the third trimester may have latent effects on cognitive development that emerge only as cognitive load increases over time [86]. This is a good example of how the effects of early childhood can alter the manifestation of brain dysfunctions, supporting emerging frameworks such as the “multiple-hit” hypothesis, which propose that neurodevelopmental disorders arise not from a single insult but from the interaction of multiple environmental and genetic risk factors across time. It is hypothesized that the rise in childhood immune and neurodevelopmental disorders reflects increasing exposure to environmental stressors during critical periods of development, which may lead to disease expression in genetically susceptible individuals [100]. According to this model, maternal immune activation during pregnancy serves as an early priming event that sensitizes the fetal brain to later-life challenges—such as psychosocial stress, drug exposure, or additional immune insults—supporting the view that cumulative exposures are key contributors to the etiology of disorders like schizophrenia and autism spectrum disorder [100]. Microglial priming is considered a key downstream effect of maternal immune activation, representing a pivotal mechanism by which early-life inflammatory exposure increases vulnerability to later environmental insults [101]. This model is further supported by animal studies demonstrating that MIA alone may not be sufficient to produce the full spectrum of behavioral or neuropathological changes, but serves to sensitize the developing brain to later-life environmental insults. For example, mice exposed to poly(I:C) in utero show exacerbated neuropathology with functional impairments when subjected to sub-chronic stress during peripubertal development [102] or double exposure of VPA and poly(I:C) at the critical timepoints mimicking behavioral features of ASD more robustly [103] than single-hit models alone. While this environmentally focused multiple-hit model emphasizes temporal accumulation of risk, it aligns conceptually with the classical two-hit hypothesis, in which a first “hit” (typically genetic) creates a vulnerable background, and a second (often environmental) event triggers the disease phenotype [104]. Together, these findings support the view that neurodevelopmental disorders most often arise from a complex interplay between genetic susceptibility and multiple environmental stressors acting across time. Rather than isolated triggers, it is the convergence and accumulation of insults—especially during critical periods of brain development—that ultimately determine disease risk and trajectory.

#### Viral infections

Viral infections have been suggested to be one of the environmental factors associated with neurodevelopmental disorders. Many viruses, such as influenza, herpesviruses 1 and 2, cytomegalovirus, Epstein-Barr virus, retrovirus, coronavirus, Zika virus and Rubella have recently been linked to neurodevelopmental disorders [105–109], but further studies are necessary to confirm these findings and explore the mechanisms involved. Viruses can interfere with normal brain maturation directly or through immune mediators such as cytokines or chemokines. In a case-control human study, multiple infections during pregnancy were associated with an increased risk of delivering a child with ASD. ASD risk was statistically similar for mothers who received treatment for their infection and for mothers who did not treat their infection [110], emphasising the importance of prevention. Increased maternal immune activation in rodents [87, 100] induced by viral infection at a specific time during CNS development has been shown to increase the prevalence of both ASD- or schizophrenia-like symptoms on the offspring. In 2012 Lee et al. showed that P2X7 plays a crucial role in the inflammatory response of the virus-recognised innate immune cells at the early stage of infection. P2X7-deficient, P2X7-inhibitor treated, caspase-1 deficient or ATP insufficient mice showed increased survival in a virus-induced high mortality respiratory distress syndrome [111].

#### Implications of SARS-CoV-2 infection on neurodevelopment

Growing evidence implicates the P2X7R/NLRP3/IL-1β axis may be involved in the immune dysregulation caused by the SARS-CoV-2 infection [112, 113], which is associated with neurological and neuropsychiatric consequences [114]. In a detailed human study, Lécuyer et al. showed that the P2X7- NLRP3 signalling pathway might play a significant role in viral replication in addition to inflammation. Increased NLRP3 expression was found independently of P2X7 in SARS-CoV-2 target host cells derived from lung autopsies of patients with severe coronavirus disease (COVID-19). This NLRP3 inflammasome activation dictates the permissiveness of epithelial cells to SARS-CoV-2 infection. P2X7 modulates SARS-CoV-2 replication through NLRP3 inflammasome activation [113]. Elevated soluble P2X7 concentrations in the plasma of COVID-19 patients correlate with disease severity and CRP levels [112]. Similarly, elevated serum P2X7 in early COVID-19 predicts adverse clinical outcomes, suggesting sP2X7 may be a useful disease progression marker [115]. Morbidity and mortality in COVID-19 are partly due to the inflammatory response [116]. Pregnant women experience more severe disease [117], and several studies suggest vertical transmission occurs [118, 119]. However, whether SARS-CoV-2 is directly vertically transmitted remains unclear [88]. Although the infection-associated inflammatory conditions during fetal development can potentially result in long-term abnormalities in the offspring [120]. Inflammatory and thrombotic lesions have been observed in the placenta of infected mothers [121]. Shuffrey et al. showed that birth during the pandemic, but not necessarily in-utero SARS-CoV-2 exposure, was linked to neurodevelopmental differences at six months [122]. However, this study has limitations and only covers up to six months of age, when few neurodevelopmental disorders are typically identified. In a prospective cohort study, Ayed et al. (2022) found that 90% of the infants born to mothers with SARS-CoV-2 infections during pregnancy had favourable outcomes, and only 10% showed developmental delays, which is similar to the general population, but the lack of precise definition makes comparison difficult. While most pregnant women in this study were infected during their third trimester, the risk of developmental delays (especially in motor skills) around 10–12 months is higher when infection occurs in the first or second trimester [123]. According to a recent meta-analysis, the evidence on the potential impact of prenatal or neonatal exposure to SARS-CoV-2 infection on early infant developmental outcomes is limited and conflicting. Larger cohorts are needed to examine ages over one year [124]. Since signs of developmental disorders often appear after 2–3 years of age, large-scale human studies of maternal COVID and neurodevelopmental disorders in the offspring remain to be conducted. A recent study by Erdogan et al. demonstrated that male rats exposed to the SARS-CoV-2 spike protein exhibited a higher rate of autism-like behaviours, gliosis, neuronal cell death in the hippocampus and cerebellum, and altered levels of MDA, TNF-α, IL-17, NF-κB, lactate, and BDNF. These findings suggest a potential link between prenatal exposure to the COVID-19 spike protein and neurodevelopmental problems [125].

#### The impact of Zika virus infection on neurodevelopment

ZIKV is a single-stranded RNA virus belonging to the family Flaviviridae and has the speciality to perturb normal brain development. The most known consequence of ZIKV infection in pregnant mothers is microcephaly in the offspring [126] induced by impaired expression of genes involved in cell replication and apoptosis [127]. In affected cases, the viral genome and antibodies were present in the cerebrospinal fluid of neonates [128], with histopathological changes observed, including calcification, necrosis, neuronophagy, gliosis, and inflammatory infiltrates in brain samples from fatal microcephaly cases [129]. In animal models, differences in neuronal arrangement within the cerebral cortex and disorganised neuronal layering [130], hippocampal dysplasia, alpha motoneuron loss, cerebellar malformations and impaired adult neurogenesis [131] of offspring from ZIKV-treated dams have been observed. These brain abnormalities are closely linked to the alterations seen in neurodevelopmental disorders [132], like delays in neuronal maturation and altered brain connectivity suggesting a causal link between prenatal Zika infection and the later development of ASD [108, 133] or schizophrenia [105]. In a study, P2X7 receptor expression increased in the brain of Zika virus-infected mice compared to the control group [134]. P2X7 increased hippocampal damage in CA1/CA2 and CA3 regions and had been linked to increased neurodegeneration, neuroinflammation and impaired motor performance in infected mice. Interestingly, wild-type mice were more effective at controlling viral load compared to P2X7 receptor-deficient mice. These findings suggest that ATP-P2X7 receptor signalling contributes to neuronal loss, neuroinflammation, and related brain abnormalities, while also involved in the antiviral response in the brain of ZIKV-infected mice [134].

#### Animal models of virus-induced maternal immune activation

To induce maternal immune activation, poly(I:C) administration to pregnant mice and rats has been widely used. These preclinical model systems help investigate the underlying pathophysiological mechanisms at the molecular, cellular and behavioural levels. Poly(I:C) is a synthetic analogue of viral double-stranded RNA and acts as an agonist for the TLR3 receptor. TLR3 generates a TRIF-dependent response by recruiting TRIF to the cytoplasmic domain, which interacts with RIP1, TRAF6, TBK1, and TRAF3, resulting in the activation of MAP kinases and the IKK complex [135]. These processes lead to the activation of ERK, JNK, IRF3, p38 and NF-κB. Nuclear translocation of ERK, JNK and p38 occurs, which activates the transcription factor AP-1, leading to further translocations (NF-κB, IRF-3 and IRF-7) to the nucleus. AP-1 and NF-κB bind to cytokine gene promoters, while IRF-3, IRF-7, and NF-κB target chemokine gene promoters, inducing their transcription [135]. Maternal cytokines and chemokines then induce immune activation, leading to systemic inflammation. Some maternal chemokines and cytokines act directly on the fetal brain. In some cases, maternal cytokine-producing cells (e.g. Th cells) themselves cross the placenta or activate effectors that can either release agents that can indirectly influence fetal development by regulating placental function or by inducing the production of additional mediators that are transported into the fetus or act directly in the fetus. Maternal cytokines may even enhance the fetus’ own cytokine production by inducing transplacental signalling pathways, which affect the developing brain. Subsequent to the placenta, the fetal BBB serves as a second barrier and demonstrates most of the barrier characteristics of the adult BBB. In humans, this barrier has been shown to be functionally active as early as the 8th week of gestation [136]; in animal models, analogous processes are assumed but occur on a different developmental timeline.

Poly(I:C)-based MIA models in mice reproduce a range of behavioral and neuroanatomical alterations relevant to neurodevelopmental disorders. Offspring frequently exhibit deficits in social interaction, increased repetitive behaviors, and impairments in sensorimotor gating, mirroring core features of autism spectrum disorder [137–139]. Typical schizophrenia-related alterations induced by poly(I:C) mimicking negative symptoms are deficits in social interaction, anhedonic behavior and cognitive impairments, such as reduced cognitive flexibility [70, 140, 141]. Importantly, Mueller et al. (2021) demonstrated that even under conditions of genetic homogeneity, MIA-exposed offspring can diverge into distinct subgroups with dissociable behavioral, transcriptional, brain network, and immunological profiles, indicating substantial interindividual variability in response to the same prenatal immune challenge [142].

These behavioral abnormalities are accompanied by widespread changes at the neuroanatomical and synaptic level. Reductions in Purkinje cell numbers within the cerebellar vermis, particularly in lobules VI and VII, have been observed in mice following maternal poly(I:C) treatment [137, 138]. These cerebellar alterations are significant given the cerebellum’s role in motor coordination and cognitive functions. Cortical disorganization and altered cortical lamination patterns [143, 144] — which may underlie the social and cognitive deficits characteristic of ASD—as well as volumetric reductions in the amygdala and hippocampus in rodent studies [145, 146], have been documented. These regions are integral to emotion processing and memory, respectively. Additionally, anomalies of the brain vasculature have also been reported [147]. In the context of schizophrenia, poly(I:C)-based animal models reveal patient-relevant neuroanatomical alterations, including reduced hippocampal volumes, region-specific gray and white matter changes [148], ventricular enlargement [148], and decreased densities of parvalbumin-expressing interneurons and perineuronal nets in the basolateral amygdala [148], collectively reflecting widespread neurodevelopmental disruption. Synaptic abnormalities have been implicated in the pathophysiology of both ASD and schizophrenia. Poly(I:C) studies report reduced dendritic spine density and arborisation in the cortex and hippocampus [70, 149], along with decreased expression of key postsynaptic proteins in excitatory hippocampal synapses during pubescence, such as PSD95 and SynGAP [150], indicating impaired organization and strengthening of excitatory receptor complexes and associated signaling proteins during synaptic maturation and connectivity. These changes are accompanied by excitation/inhibition imbalance in cortical circuits [151, 152], and ultrastructural synaptic abnormalities, including increased number of malformed synaptosomes [137] indicating disrupted synaptic architecture.

Altogether, poly(I:C)-induced activation of TLR3 in rodent models effectively mimics viral maternal immune activation and provides mechanistic insight into how maternal cytokines, chemokines, and placental signaling disturbances can influence fetal brain development during critical neurodevelopmental windows.

#### Bacterial infections

Maternal bacterial infection during pregnancy may also produce long-term alterations in the structure and function of the fetal brain [95, 99] and are linked to psychotic disorders, particularly in males, with severity of infection playing a role. The effect of multisystemic bacterial infection is almost twice that of less severe local bacterial infection [153]. Bacterial infections, especially asymptomatic UTIs, are often left untreated in antenatal care, potentially posing a significant threat to healthy fetal development. Animal studies show prenatal lipopolysaccharide exposure, mimicking gram-negative bacterial infection, induces autistic-like behaviours [154, 155]. LPS acting on the TLR4 receptor leads to the formation of a receptor complex consisting of dimerised TLR4 and MD-2. This activates two signalling pathways: a MyD88/Mal-dependent pathway for early NF-κB activation, and a TRIF/TRAM-dependent pathway for late NF-κB and IRF3 activation, through which the induction of inflammasome, cytokines, chemokines and other transcription factors starts [156].

In humans, maternal urinary tract infections are common during pregnancy due to immune, hormonal, anatomical and habitual changes. Infections that are not treated with antibiotics are associated with an increased risk of mental retardation or developmental delay in infants [157]. Neonatal sepsis is also associated with an increased risk of autism diagnosis in both preterm and in-term children [158]. P2X7 activation, confirmed in animal sepsis studies [159] leads to recruitment of inflammatory cells in the cerebral vasculature, destruction of the BBB, activation of microglial cells in the brain, apoptosis of brain cells and other damage processes leading to diffuse brain damage as a result of secondary infection in other parts of the body [160]. At the same time, the P2X7 receptor is required for the development of the sepsis-associated inflammatory response throughout the body [161]. Martínez-García et al. showed that, in addition to its more known effects, P2X7 may also play a role in the generation of mitochondrial dysfunction during sepsis, leading to impaired NLRP3 and IL-1β activation, which is involved in the development of an immunodeficient status in patients with sepsis and in the associated mortality. Blocking the P2X7 seems to be protective against sepsis pathogenesis [159, 162], and organ dysfunction [163–165]. Pharmacological inhibition or genetic deletion of P2X7 receptor attenuates the production of IL-1β, reduces neuroinflammation and prevents cognitive impairments in sepsis-surviving animals [166]. However, surprisingly there are a few studies describing a protective role of P2X7 by decreasing bacterial dissemination as well with increased mortality in P2rx7^−/−^ mice [167, 168]. Fialho et al. also draw attention to the potential protective effect of P2X7 in neonatal sepsis and that therapeutic targeting of purinergic receptors should respect the pathogen-controlling beneficial effects of P2X7 in addition to its excessive inflammatory response-inducing effects [161]. However, taking into account the relevant literature data, it can be concluded that the role of P2X7 in sepsis, the anti-inflammatory feature of P2X7 antagonists and the hypo-inflammatory nature of neonatal immune responses make P2X7 a potential target for neonatal sepsis as an adjunct to antibiotic therapy, provided that pathogen elimination as a result of antibiotic treatment is adequately supported by laboratory tests. As surviving neonatal sepsis has potential to cause a range of neurocognitive impairments, successful clinical implementation of P2X7 inhibition may reduce the development of neurodevelopmental disorders in affected children.

#### Prenatal Toxoplasma gondii infection

Toxoplasma gondii, a major cause of human spontaneous abortions [169], primarily infects the brain, targeting neurons. The parasite evades immune responses for persistent infection [170]. Intrauterine infection risks fetal brain defects and subsequent neuropsychiatric disorders [171, 172]. In infected mouse neural progenitor cells, P2X7 receptor expression increases, alongside altered ectonucleotidase activity, augmenting ATP/ADP hydrolysis. This suggests extracellular ATP activates P2X7 on microglia, and continued activation of P2X7 receptors by ATP during chronic infection has been proposed as a mechanism for the elimination of T. gondii [173]. Moreira-Souza and Coutinho-Silva’s review summarises the role of purinergic signalling in T. gondii infection, highlighting in vitro studies demonstrating P2X7 activation’s microbicidal effect in phagocytic and parasite control in non-phagocytic cells [174].

### Autoimmune diseases

There is evidence to assume that the inflammatory environment in pregnant mothers can be triggered by damage to intrinsic mechanisms in addition to external stimuli, creating a similar inflammatory environment and inducing signalling pathways that can lead to the development of a potential neurodevelopmental disorder [175]. During autoimmunity, a specific adaptive immune response is mounted against self antigens and abnormal IgG can cross the placenta [176], potentially causing fetal issues. Maternal autoimmune diseases are linked to developmental disorders [177, 178], and maternal autoantibodies increase ASD risk [179, 180]. Maternal hypothyroidism, especially in the first trimester, is also associated with ASD [181] disrupting neuronal migration. Even a moderate transient period of first-trimester maternal hypothyroxinemia, not necessarily associated with TSH elevation, disrupts the migration of neurons to the cortical layers at the onset of neurogenesis in rats [182]. In autoimmune encephalitis, the immune system attacks brain cells, causing inflammation and autism-like traits [183]. In rodents, maternal autoantibodies administered during gestation migrated to the cortical parenchyma, altering coronal development and neuritogenesis, resulting in fewer mature dendritic spines in adult mice [184, 185], ) and displayed ASD-like behaviours including increased repetitive behaviours and altered social interactions [186]. The P2X7 receptor has also been implicated in autoimmune encephalitis as an early exacerbator of CNS inflammation [187], and fuels autoimmunity via proinflammatory cytokine release [188]. P2X7 inhibitors were in clinical phase II trials in the treatment of some autoimmune diseases [189, 190], so it would be important to further investigate the role of P2X7 during pregnancy and its potential external modulation during this critical period.

## Translational relevance of the P2X7/NLRP3/IL-1β pathway in neurodevelopment

### Experimental evidence linking maternal inflammation to neurodevelopmental outcomes

Growing evidence suggests maternal inflammation is the key mediator of the increased risk of developmental abnormalities linked to environmental conditions mentioned earlier. Recent studies have extensively examined how pregnancy-related inflammation is connected to neurological disorders, using experimental data from animal studies to assess its impact on brain development. Activated maternal immunity elevates extracellular ATP in developing neural structures [138, 191]. Beamer et al. found that increased extracellular ATP, via P2X7 receptors, alters action potential threshold and maximum firing frequency, thereby modulating neonatal subplate neuron activity [69]. Recently, we showed that MIA-induced proinflammatory cytokine- and chemokine-level increase in maternal plasma (IL-1β, RANTES, MCP-1) and fetal brain (IL-1β, IL-2, IL-6, MCP-1) is regulated by the P2X7 - NLRP3 pathway in a mouse model of ASD [137]. Genetic or pharmacological inhibition of P2X7 receptor resulted in the absence of autism-like behaviours and cytokine level alterations in young adulthood suggesting that P2X7 alone is sufficient and also necessary for the development of autistic features in mice during neuronal development [137, 138]. Similarly, maternal treatment with NLRP3 antagonist or a neutralising IL-1β antibody during pregnancy also counteracted the development of autistic phenotype in offspring mice [137]. Coadministration of IL-1 receptor antagonist with LPS seems to alleviate LPS-induced placental inflammation and fetal brain damage (forebrain white matter and motor behavioural alterations) in offspring rats as well [192]. Prenatal poly(I:C) exposure elevates the P2X7/NLRP3/IL-1β inflammatory pathway in the nucleus accumbens of female juvenile rats [193]. Both LPS and poly(I:C) treatment induced the expression and activation of NLRP3 and IL-1β in the brain of offspring rats showing schizophrenia-like behaviours [194] In vitro studies show higher baseline and LPS-induced Caspase-1, NLRP3, and secreted IL-1β levels in PBMCs from individuals with ASD [195]. Inhibition of the NLRP3 with MCC950 blocks both the NLRP3-mediated and the baseline IL-1β production and decreased caspase-1 activity in the PBMC cultures [195]. Postnatal human studies show that children and adults with ASD have increased plasma IL-1β [196]. These results suggest that elevated IL-1β expression in the brain may be a long-term product of sustained inflammation. Elevated IL-1β in the circulation shortly after preterm birth has been associated with an increased risk of neurodevelopmental disorders at 24 months of age in humans [197]. In term/near-term newborns, elevated systemic and cerebrospinal IL-1β on the first days of life has been linked to impaired cerebral metabolism and developmental delay at 30 months of age [198, 199]. Kelly et al. support the clinical utilization of interleukin-1 receptor antagonists for infants at greatest risk of perinatal inflammation and neurodevelopmental impairment [200]. While the exact mechanisms of P2X7/NLRP3/IL-1β pathway activation in pathological brain development remain unclear, some morphological differences have been described. In pathological conditions P2X7 activation also causes abnormalities in dendritic morphology and deficits in dendritic outgrowth, based on an ex vivo study using a poly(I:C) mouse model, and the identical treatment leads to behavior abnormalities on young-adilt offspring [70]. Fetsko et al. showed that CNS-specific expression of IL-1β interferes with BBB development by disrupting Wnt/β-catenin signalling in brain endothelial cells during neurovascular development, leading to a significant reduction in CNS angiogenesis and barrier formation [61].

### Genetic variants of the PX7 receptor and their potential role in neurodevelopmental susceptibility

Given the critical roles of P2X7, NLRP3, and IL-1β in brain development and neuroinflammatory processes, genetic variations affecting these pathways may influence neurodevelopmental outcomes, particularly under inflammatory conditions. Several single-nucleotide polymorphisms (SNPs) in the P2RX7 gene are known to alter receptor function, influencing pore formation, ATP sensitivity, or cytokine release. Among these, two well-studied gain-of-function variants are rs2230912 (Gln460Arg) and rs1718119 (Ala348Thr). The rs2230912 variant has been associated with increased P2X7-mediated IL-1β release and heightened inflammatory responses [201, 202]. Clinical studies have linked it to rapid cycling in bipolar disorder type 1, where P2RX7 expression was found to be modulated by sleep deprivation [203] and also to major depression [204]. Similarly, rs1718119 has been associated with increased cytokine release, enhanced pore activity, and elevated receptor function [202]. This variant has also been linked to increased depression severity [205] and may interact with rs2230912 in influencing susceptibility to affective disorders, including rapid cycling in bipolar disorder [203]. Moreover, a recent proteome-wide association study identified P2RX7 among 25 brain-expressed proteins causally linked to depression, providing additional support for the involvement of this receptor in affective disorders [206]. Other variants, including rs1653625, rs3751143, rs208294 and rs7958311, have also been associated with depressive disorders [205, 207, 208]. However, no data are currently available on their association with schizophrenia or ASD. Similarly, no significant association between P2RX7 polymorphisms and schizophrenia was detected in a Danish cohort [209]. The authors emphasise that the possibility of a weak or subtype-specific genetic contribution cannot be excluded due to limited statistical power and low variant frequencies. Despite biological plausibility and shared inflammatory mechanisms, no P2RX7 variants have shown consistent, genome-wide significant associations with schizophrenia yet [209, 210]. Similarly, no P2RX7 variants have been directly associated with autism spectrum disorder (ASD) in genetic studies to date. Nevertheless, experimental models have demonstrated that P2X7-mediated neuroinflammation during prenatal development can contribute to ASD-like phenotypes, highlighting the need for future research into whether P2RX7 genetic variation modulates ASD risk, particularly under conditions of maternal immune activation.

Although no specific NLRP3 or IL1B gene variants have been directly associated with schizophrenia, autism, or depression, polymorphisms in these genes have been implicated in broader neuroinflammatory processes. Gain-of-function mutations in NLRP3 can lead to excessive IL-1β production and are primarily linked to autoinflammatory syndromes, but the resulting chronic inflammation may also impact neurodevelopmental processes [211]. While these mutations are primarily associated with autoinflammatory syndromes, the resultant chronic inflammation could conceivably impact neurodevelopmental processes, although direct links to specific neurodevelopmental disorders require further investigation to elucidate these potential connections.

As described earlier, P2X7/NLRP3/IL-1β pathway plays essential roles in both embryonic and adult neurodevelopment—including axonal growth, cell cycle regulation, and neuroinflammatory modulation. A gain-of-function variant may exaggerate ATP-induced inflammasome activation during critical developmental windows, potentially shifting the delicate balance of P2X7 activity from physiological neurodevelopmental regulation to pathological inflammation. While no P2RX7 variant has been directly linked to autism or schizophrenia, the convergence of genetic predisposition and environmental immune challenges—such as maternal infection—could intensify neuroinflammatory responses, contributing to altered neuronal migration, synaptic formation, or neurogenesis. This gene–environment interaction model provides a plausible framework for understanding the heterogeneity of neurodevelopmental outcomes following prenatal immune activation and highlights the importance of studying genetic susceptibility in conjunction with temporally specific inflammatory exposures.

### Targeting the P2X7/NLRP3/IL-1β axis: therapeutic strategies, ethical challenges, and population relevance

The P2X7/NLRP3/IL-1β signalling axis has emerged as a promising therapeutic target in various neurological and psychiatric disorders characterized by neuroinflammation. Preclinical studies have demonstrated that pharmacological inhibition of this pathway may ameliorate behavioral and neuropathological symptoms in models of depression [212], multiple sclerosis [213, 214], Alzheimer’s disease [215, 216], and traumatic brain injury [217]. Selective P2X7 antagonists such as JNJ-47965567 and NLRP3 inhibitors like MCC950 have shown efficacy in reducing IL-1β production, decreasing neuroinflammation, and improving behavioral outcomes in animal studies [137, 211, 218]. In maternal immune activation (MIA) models, blockade of P2X7, NLRP3, or IL-1β signaling during pregnancy mitigated neurodevelopmental and behavioral alterations in offspring [137], supporting the therapeutic relevance of this pathway in prenatal contexts. Although JNJ-47965567 has not been tested in pregnant women, preclinical studies have reported it to be non-toxic and well-tolerated, supporting its safety profile in general use [219]. Nonetheless, any pharmacological agent that interferes with immune signaling carries a theoretical risk of off-target effects, particularly during sensitive developmental windows such as pregnancy.

However, the potential use of immunomodulatory agents during pregnancy raises substantial ethical concerns. The P2X7 receptor, NLRP3 inflammasome, and IL-1β also play essential physiological roles in normal neurodevelopment, including their contributions to neural differentiation, synaptic plasticity, and immune surveillance. Consequently, selective modulation that preserves baseline activity while attenuating pathological hyperactivation is critical to prevent unintended interference with developmental processes. This principle is especially important in pregnancy, where therapeutic strategies must ensure both maternal safety and fetal neurodevelopmental integrity. Therefore, therapeutic interventions must aim to restore immune balance rather than achieve complete inhibition.

The scope of the problem further underlines the urgency of developing targeted, safe interventions. Autism spectrum disorder affects approximately 1 in 36 children [220], while the lifetime prevalence of schizophrenia is estimated at around 1 in 300 people globally [221]. Growing epidemiological evidence indicates that a significant proportion of these neurodevelopmental disorders may be linked to prenatal environmental factors, including maternal infections, autoimmune conditions, and inflammatory exposures [90]. This highlights the importance of understanding how inflammation-driven pathways—such as P2X7/NLRP3/IL-1β—mediate these effects and how they might be safely modulated in future therapeutic approaches. Taken together, these considerations suggest that while the P2X7/NLRP3/IL-1β pathway holds great promise as a therapeutic target, any clinical application must be grounded in precision, timing, and ethical responsibility. Further research is warranted to develop targeted strategies that normalize immune activity without compromising essential developmental functions, paving the way for safe preventive or therapeutic interventions during pregnancy.

## Conclusion

Neurodevelopmental diseases have been linked to the activation of the P2X7 receptor, inducing the assembly of the NLRP3 inflammasome and the subsequent release of IL-1β and other proinflammatory mediators. The review of the relevant literature on the role of immune activation through the P2X7/NLRP3/IL-1β pathway in the effects of infection and neuronal inflammation on brain development highlights the need to further investigate the effects of P2X7 inhibition in various neurodevelopmental conditions and to translate these findings into clinical applications. Blocking components of this pathway presents a promising therapeutic approach to mitigate neurodevelopmental damage caused by perinatal inflammation, particularly during critical periods such as the first and second trimesters. However, there are two key aspects of future approaches to eliminate: First, it is important to evaluate the protective role of P2X7 in defence mechanisms against microorganisms during all prenatal conditions, alongside its detrimental effects that lead to significant neurodegeneration and neuroinflammation. Second, any therapeutic intervention should be designed to avoid disrupting the P2X7-mediated mechanisms that are essential for normal physiological development. Overall, targeting the P2X7/NLRP3/IL-1β pathway may offer new options for prevention and treatment, emphasising the balance between managing inflammatory responses to protect fetal brain development and maintaining necessary immune functions.

## Data Availability

No datasets were generated or analysed during the current study.

## References

[1] Spaans F, et al. Danger signals from ATP and adenosine in pregnancy and preeclampsia. Hypertension. 2014;63(6):1154–60. 24688119 10.1161/HYPERTENSIONAHA.114.03240

[2] Rodrigues RJ, Marques JM, Cunha RA. Purinergic signalling and brain development. Semin Cell Dev Biol. 2019;95:34–41. 30529149 10.1016/j.semcdb.2018.12.001

[3] Burnstock G. P2X ion channel receptors and inflammation. Purinergic Signal. 2016;12(1):59–67. 26739702 10.1007/s11302-015-9493-0 PMC4749528

[4] Pelegrin P. P2X7 receptor and the NLRP3 inflammasome: Partners in crime. Biochem Pharmacol. 2021;187:114385. 33359010 10.1016/j.bcp.2020.114385

[5] Di Virgilio F, et al. The P2X7 Receptor in Infection and Inflammation. Immunity. 2017;47(1):15–31. 28723547 10.1016/j.immuni.2017.06.020

[6] Yin W, Godbout JP, Sheridan JF. Chap. 7 - Interleukin-1 beta in psychosocial stress. Stress: immunology and inflammation. Academic; 2024;53–63. G. Fink, Editor.

[7] Kaneko N, et al. The role of interleukin-1 in general pathology. Inflamm Regeneration. 2019;39(1):12. 10.1186/s41232-019-0101-5 PMC6551897 31182982

[8] Sutton CE, et al. Interleukin-1 and IL-23 induce innate IL-17 production from γδ T cells, amplifying Th17 responses and autoimmunity. Immunity. 2009;31(2):331–41. 19682929 10.1016/j.immuni.2009.08.001

[9] Deverman BE, Patterson PH. Cytokines and CNS development. Neuron. 2009;64(1):61–78. 19840550 10.1016/j.neuron.2009.09.002

[10] Hewett SJ, Jackman NA, Claycomb RJ. Interleukin-1β in central nervous system injury and repair. Eur J Neurodegener Dis. 2012;1(2):195–211. 26082912 PMC4465544

[11] Fields RD. Nonsynaptic and nonvesicular ATP release from neurons and relevance to neuron-glia signaling. Semin Cell Dev Biol. 2011;22(2):214–9. 21320624 10.1016/j.semcdb.2011.02.009 PMC3163842

[12] Koizumi S. Synchronization of Ca2 + oscillations: involvement of ATP release in astrocytes. Febs J. 2010;277(2):286–92. 19895581 10.1111/j.1742-4658.2009.07438.x

[13] George J, et al. Different danger signals differently impact on microglial proliferation through alterations of ATP release and extracellular metabolism. Glia. 2015;63(9):1636–45. 25847308 10.1002/glia.22833

[14] Bodin P, Burnstock G. Purinergic signalling: ATP release. Neurochem Res. 2001;26(8–9):959–69. 11699948 10.1023/a:1012388618693

[15] Chen YH, et al. Extracellular ATP is a homeostatic messenger that mediates Cell-Cell communication in physiological processes and psychiatric diseases. Biol Psychiatry. 2025;97(1):41–53. 38679359 10.1016/j.biopsych.2024.04.013

[16] Giuliani AL, Sarti AC, Di Virgilio F. Ectonucleotidases in acute and chronic inflammation. Front Pharmacol. 2020;11:619458. 33613285 10.3389/fphar.2020.619458 PMC7887318

[17] Taruno A. ATP release channels. Int J Mol Sci. 2018;19(3):808. 29534490 10.3390/ijms19030808 PMC5877669

[18] Burnstock G, Kennedy C. P2X receptors in health and disease. Adv Pharmacol. 2011;61:333–72. 21586364 10.1016/B978-0-12-385526-8.00011-4

[19] North RA. Molecular physiology of P2X receptors. Physiol Rev. 2002;82(4):1013–67. 12270951 10.1152/physrev.00015.2002

[20] Rodrigues RJ, Tomé AR, Cunha RA. ATP as a multi-target danger signal in the brain. Front Neurosci. 2015;9:148. 25972780 10.3389/fnins.2015.00148 PMC4412015

[21] Sperlágh B, Illes P. P2X7 receptor: an emerging target in central nervous system diseases. Trends Pharmacol Sci. 2014;35(10):537–47. 25223574 10.1016/j.tips.2014.08.002

[22] Bidula S, et al. Positive allosteric modulation of P2X7 promotes apoptotic cell death over lytic cell death responses in macrophages. Cell Death Dis. 2019;10(12):882. 31767863 10.1038/s41419-019-2110-3 PMC6877589

[23] Purohit R, Bera AK. Pannexin 1 plays a pro-survival role by attenuating P2X7 receptor-mediated Ca2 + influx. Cell Calcium. 2021;99:102458. 34479067 10.1016/j.ceca.2021.102458

[24] Crespo Yanguas S, et al. Pannexin1 as mediator of inflammation and cell death. Biochimica et Biophysica Acta (BBA). - Mol Cell Res. 2017;1864(1):51–61. 10.1016/j.bbamcr.2016.10.006 PMC5693326 27741412

[25] Weilinger NL, et al. Pannexin-1 opening in neuronal edema causes cell death but also leads to protection via increased microglia contacts. Cell Rep. 2023;42(10):113128. 37742194 10.1016/j.celrep.2023.113128 PMC10824275

[26] Boyce AKJ, Swayne LA. P2X7 receptor cross-Talk regulates ATP-induced pannexin 1 internalization. Biochem J. 2017;474(13):2133–44. 28495860 10.1042/BCJ20170257

[27] Ruan Z, et al. Structures of human pannexin 1 reveal ion pathways and mechanism of gating. Nature. 2020;584(7822):646–51. 32494015 10.1038/s41586-020-2357-y PMC7814660

[28] Mim C, Perkins G, Dahl G. Structure versus function: are new conformations of pannexin 1 yet to be resolved? J Gen Physiol, 2021;153(5). 10.1085/jgp.202012754 PMC8042604 33835130

[29] Baroja-Mazo A, Barberà-Cremades M, Pelegrín P. The participation of plasma membrane hemichannels to purinergic signaling. Biochim Et Biophys Acta - Biomembr. 2013;1828(1):79–93. 10.1016/j.bbamem.2012.01.002 22266266

[30] Li D, Wu M. Pattern recognition receptors in health and diseases. Signal Transduct Target Therapy. 2021;6(1):291. 10.1038/s41392-021-00687-0 PMC8333067 34344870

[31] Acioglu C, Heary RF, Elkabes S. Roles of neuronal toll-like receptors in neuropathic pain and central nervous system injuries and diseases. Behav Immun. 2022;102:163–78. Brain. 10.1016/j.bbi.2022.02.016 35176442

[32] Kielian T. Toll-like receptors in central nervous system glial inflammation and homeostasis. J Neurosci Res. 2006;83(5):711–30. 16541438 10.1002/jnr.20767 PMC2440498

[33] Yang J, Yan H. TLR5: beyond the recognition of Flagellin. Cell Mol Immunol. 2017;14(12):1017–9. 29151579 10.1038/cmi.2017.122 PMC5719140

[34] Miyake K, et al. Nucleic acid sensing by Toll-Like receptors in the endosomal compartment. Front Immunol. 2022;13:941931. 35812450 10.3389/fimmu.2022.941931 PMC9259784

[35] Zhang Z, et al. Structural analyses of Toll-like receptor 7 reveal detailed RNA sequence specificity and recognition mechanism of agonistic ligands. Cell Rep. 2018;25(12):3371–e33815. 30566863 10.1016/j.celrep.2018.11.081

[36] Semple C, et al. Polymorphisms in the P2X7 receptor, and differential expression of Toll-like receptor-mediated cytokines and defensins, in a Canadian Indigenous group. Sci Rep. 2019;9(1):14204. 31578370 10.1038/s41598-019-50596-0 PMC6775093

[37] Zhang W-J, et al. NLRP3 inflammasome: A key contributor to the inflammation formation. Food Chem Toxicol. 2023;174:113683. 36809826 10.1016/j.fct.2023.113683

[38] Yang Y, et al. Recent advances in the mechanisms of NLRP3 inflammasome activation and its inhibitors. Cell Death Dis. 2019;10(2):128. 30755589 10.1038/s41419-019-1413-8 PMC6372664

[39] Zangiabadi S, Abdul-Sater AA. Regulation of the NLRP3 inflammasome by posttranslational modifications. J Immunol. 2022;208(2):286–92. 35017218 10.4049/jimmunol.2100734

[40] Karmakar M, et al. Neutrophil P2X7 receptors mediate NLRP3 inflammasome-dependent IL-1β secretion in response to ATP. Nat Commun. 2016;7:10555. 26877061 10.1038/ncomms10555 PMC4756306

[41] Próchnicki T, Mangan MS, Latz E. Recent insights into the molecular mechanisms of the NLRP3 inflammasome activation. F1000Res, 2016;5. 10.12688/f1000research.8614.1 PMC4963208 27508077

[42] Swanson KV, Deng M, Ting JP. The NLRP3 inflammasome: molecular activation and regulation to therapeutics. Nat Rev Immunol. 2019;19(8):477–89. 31036962 10.1038/s41577-019-0165-0 PMC7807242

[43] Groß CJ, et al. K(+) Efflux-Independent NLRP3 inflammasome activation by small molecules targeting mitochondria. Immunity. 2016;45(4):761–73. 27692612 10.1016/j.immuni.2016.08.010

[44] Tschopp J, Schroder K. NLRP3 inflammasome activation: the convergence of multiple signalling pathways on ROS production? Nat Rev Immunol. 2010;10(3):210–5. 20168318 10.1038/nri2725

[45] Boucher D, et al. Caspase-1 self-cleavage is an intrinsic mechanism to terminate inflammasome activity. J Exp Med. 2018;215(3):827–40. 29432122 10.1084/jem.20172222 PMC5839769

[46] Ting JP, Willingham SB, Bergstralh DT. NLRs at the intersection of cell death and immunity. Nat Rev Immunol. 2008;8(5):372–9. 18362948 10.1038/nri2296

[47] Bockstiegel J, Engelhardt J, Weindl G. P2X7 receptor activation leads to NLRP3-independent IL-1β release by human macrophages. Cell Communication Signal. 2023;21(1):335. 10.1186/s12964-023-01356-1 PMC10666422 37996864

[48] Andrei C, et al. Phospholipases C and A2 control lysosome-mediated IL-1β secretion: implications for inflammatory processes. Proc Natl Acad Sci. 2004;101(26):p9745–9750. 10.1073/pnas.0308558101 PMC470745 15192144

[49] MacKenzie A, et al. Rapid secretion of interleukin-1β by microvesicle shedding. Immunity. 2001;15(5):825–35. 11728343 10.1016/s1074-7613(01)00229-1

[50] Qu Y, et al. Nonclassical IL-1 beta secretion stimulated by P2X7 receptors is dependent on inflammasome activation and correlated with exosome release in murine macrophages. J Immunol. 2007;179(3):1913–25. 17641058 10.4049/jimmunol.179.3.1913

[51] Shirasaki Y, et al. Real-time single-cell imaging of protein secretion. Sci Rep. 2014;4(1):4736. 24751898 10.1038/srep04736 PMC3994437

[52] Bergsbaken T, Fink SL, Cookson BT. Pyroptosis: host cell death and inflammation. Nat Rev Microbiol. 2009;7(2):99–109. 19148178 10.1038/nrmicro2070 PMC2910423

[53] Grebenciucova E, VanHaerents S. Interleukin 6: at the interface of human health and disease. Front Immunol. 2023;14:1255533. 37841263 10.3389/fimmu.2023.1255533 PMC10569068

[54] Rauf A et al. Neuroinflammatory markers: key indicators in the pathology of neurodegenerative diseases. Molecules, 2022;27(10). 10.3390/molecules27103194 PMC9146652 35630670

[55] Dinarello CA. Overview of the IL-1 family in innate inflammation and acquired immunity. Immunol Rev. 2018;281(1):8–27. 29247995 10.1111/imr.12621 PMC5756628

[56] Luís JP, Simões CJV, Brito RMM. The therapeutic prospects of targeting IL-1R1 for the modulation of neuroinflammation in central nervous system disorders. Int J Mol Sci. 2022;23(3):1731. 35163653 10.3390/ijms23031731 PMC8915186

[57] Sims JE, Smith DE. The IL-1 family: regulators of immunity. Nat Rev Immunol. 2010;10(2):89–102. 20081871 10.1038/nri2691

[58] Liu X, et al. Cell-Type-Specific Interleukin 1 receptor 1 signaling in the brain regulates distinct neuroimmune activities. Immunity. 2019;50(2):317–33..e6. 30683620 10.1016/j.immuni.2018.12.012 PMC6759085

[59] Huang Y, et al. Neuron-specific effects of interleukin-1β are mediated by a novel isoform of the IL-1 receptor accessory protein. J Neurosci. 2011;31(49):18048–59. 22159118 10.1523/JNEUROSCI.4067-11.2011 PMC3261076

[60] Banks WA, Kastin AJ, Broadwell RD. Passage of cytokines across the blood-brain barrier. Neuroimmunomodulation. 1995;2(4):241–8. 8963753 10.1159/000097202

[61] Fetsko AR et al. IL-1β disrupts blood-brain barrier development by inhibiting endothelial Wnt/β-catenin signaling. bioRxiv, 2024;2023.12.04.569943. 10.1016/j.isci.2024.109651 PMC11025013 38638574

[62] Ribeiro DE, et al. Purinergic receptors in neurogenic processes. Brain Res Bull. 2019;151:3–11. 30593881 10.1016/j.brainresbull.2018.12.013

[63] Burnstock G, Ulrich H. Purinergic signaling in embryonic and stem cell development. Cell Mol Life Sci. 2011;68(8):1369–94. 21222015 10.1007/s00018-010-0614-1 PMC11114541

[64] Tsao HK, Chiu PH, Sun SH. PKC-dependent ERK phosphorylation is essential for P2X7 receptor-mediated neuronal differentiation of neural progenitor cells. Cell Death Dis. 2013;4(8):e751. 23907465 10.1038/cddis.2013.274 PMC3763436

[65] Cheung KK, Chan WY, Burnstock G. Expression of P2X purinoceptors during rat brain development and their inhibitory role on motor axon outgrowth in neural tube explant cultures. Neuroscience. 2005;133(4):937–45. 15964486 10.1016/j.neuroscience.2005.03.032

[66] Ortega F, et al. Salient brain entities labelled in P2rx7-EGFP reporter mouse embryos include the septum, roof plate glial specializations and circumventricular ependymal organs. Brain Struct Funct. 2021;226(3):715–41. 33427974 10.1007/s00429-020-02204-5 PMC7981336

[67] Sebastián-Serrano Á, et al. Studying the Role of P2X7 Receptor in Axonal Growth Using In Utero Electroporation Technique. Methods Mol Biol. 2022;2510:355–66. 35776336 10.1007/978-1-0716-2384-8_20

[68] Glaser T, et al. Modulation of mouse embryonic stem cell proliferation and neural differentiation by the P2X7 receptor. PLoS ONE. 2014;9(5):e96281. 24798220 10.1371/journal.pone.0096281 PMC4010452

[69] Beamer E, Kovács G, Sperlágh B. ATP released from astrocytes modulates action potential threshold and spontaneous excitatory postsynaptic currents in the neonatal rat prefrontal cortex. Brain Res Bull. 2017;135:129–42. 29030320 10.1016/j.brainresbull.2017.10.006

[70] Mut-Arbona P, et al. Dual Role of the P2X7 Receptor in Dendritic Outgrowth during Physiological and Pathological Brain Development. J Neurosci. 2023;43(7):1125–42. 36732073 10.1523/JNEUROSCI.0805-22.2022 PMC9962779

[71] Andrejew R, et al. Post-weaning social isolation impairs purinergic signaling in rat brain. Neurochem Int. 2021;148:105111. 34171414 10.1016/j.neuint.2021.105111

[72] Miras-Portugal MT, et al. Neuronal P2X7 Receptor: Involvement in Neuronal Physiology and Pathology. J Neurosci. 2017;37(30):7063–72. 28747389 10.1523/JNEUROSCI.3104-16.2017 PMC6705729

[73] Delarasse C, et al. Neural progenitor cell death is induced by extracellular ATP via ligation of P2X7 receptor. J Neurochem. 2009;109(3):846–57. 19250337 10.1111/j.1471-4159.2009.06008.x

[74] Oliveira Á, Illes P, Ulrich H. Purinergic receptors in embryonic and adult neurogenesis. Neuropharmacology. 2016;104:272–81. 26456352 10.1016/j.neuropharm.2015.10.008

[75] Leeson HC, et al. P2X7 receptor signaling during adult hippocampal neurogenesis. Neural Regen Res. 2019;14(10):1684–94. 31169175 10.4103/1673-5374.257510 PMC6585562

[76] Csölle C, et al. Neurochemical Changes in the Mouse Hippocampus Underlying the Antidepressant Effect of Genetic Deletion of P2X7 Receptors. PLoS ONE. 2013;8(6):e66547. 23805233 10.1371/journal.pone.0066547 PMC3689833

[77] Lammert CR, et al. AIM2 inflammasome surveillance of DNA damage shapes neurodevelopment. Nature. 2020;580(7805):647–52. 32350463 10.1038/s41586-020-2174-3 PMC7788527

[78] Coutinho-Budd JC, Broihier HT. Pyroptosis takes aim at neurodevelopment. Dev Cell. 2020;53(5):498–9. 32516594 10.1016/j.devcel.2020.05.013

[79] McKenzie BA, Dixit VM, Power C. Fiery cell death: pyroptosis in the central nervous system. Trends Neurosci. 2020;43(1):55–73. 31843293 10.1016/j.tins.2019.11.005

[80] Ma L, et al. Interleukin-1 beta guides the migration of cortical neurons. J Neuroinflammation. 2014;11:114. 24950657 10.1186/1742-2094-11-114 PMC4084576

[81] Mousa A, et al. HUMAN FIRST TRIMESTER FOREBRAIN CELLS EXPRESS GENES FOR INFLAMMATORY AND ANTI-INFLAMMATORY CYTOKINES. Cytokine. 1999;11(1):55–60. 10080879 10.1006/cyto.1998.0381

[82] Giulian D, et al. Interleukin-1 is an astroglial growth factor in the developing brain. J Neurosci. 1988;8(2):709–14. 3257519 10.1523/JNEUROSCI.08-02-00709.1988 PMC6569312

[83] Striedinger K, Scemes E. Interleukin-1beta affects calcium signaling and in vitro cell migration of astrocyte progenitors. J Neuroimmunol. 2008;196(1–2):116–23. 18462808 10.1016/j.jneuroim.2008.03.014 PMC2453308

[84] Boato F, et al. Interleukin-1 beta and neurotrophin-3 synergistically promote neurite growth in vitro. J Neuroinflammation. 2011;8:183. 22200088 10.1186/1742-2094-8-183 PMC3275552

[85] Prem S, Millonig JH, DiCicco-Bloom E. Dysregulation of neurite outgrowth and cell migration in autism and other neurodevelopmental disorders. Neurodevelopmental Disorders: Employing iPSC Technologies to Define and Treat Childhood Brain Diseases, 2020;109–153. 10.1007/978-3-030-45493-7_5 32578146

[86] Kwok J, et al. Maternal infections during pregnancy and child cognitive outcomes. BMC Pregnancy Childbirth. 2022;22(1):848. 36397016 10.1186/s12884-022-05188-8 PMC9670450

[87] Knuesel I, et al. Maternal immune activation and abnormal brain development across CNS disorders. Nat Rev Neurol. 2014;10(11):643–60. 25311587 10.1038/nrneurol.2014.187

[88] Zimmer A, et al. Prenatal exposure to viral infection and neuropsychiatric disorders in offspring: A review of the literature and recommendations for the COVID-19 pandemic. Brain Behav Immun. 2021;91:756–70. 33152446 10.1016/j.bbi.2020.10.024 PMC7759331

[89] Tau GZ, Peterson BS. Normal development of brain circuits. Neuropsychopharmacology. 2010;35(1):147–68. 19794405 10.1038/npp.2009.115 PMC3055433

[90] Doi M, Usui N, Shimada S. Prenatal environment and neurodevelopmental disorders. Front Endocrinol (Lausanne). 2022;13:860110. 35370942 10.3389/fendo.2022.860110 PMC8964779

[91] Lanzone A, Ferrazzani S, Botta A. Delivery and late preterm birth. Ital J Pediatr. 2014;40(2):A1.

[92] San Martín-González N, et al. Maternal respiratory viral infections during pregnancy and offspring’s neurodevelopmental outcomes: A systematic review. Neurosci Biobehav Rev. 2023;149:105178. 37059407 10.1016/j.neubiorev.2023.105178

[93] Brown AS, et al. A.E. Bennett research award. Prenatal rubella, premorbid abnormalities, and adult schizophrenia. Biol Psychiatry. 2001;49(6):473–86. 11257233 10.1016/s0006-3223(01)01068-x

[94] Brown AS, et al. Serologic evidence of prenatal influenza in the etiology of schizophrenia. Arch Gen Psychiatry. 2004;61(8):774–80. 15289276 10.1001/archpsyc.61.8.774

[95] Sørensen HJ, et al. Association between prenatal exposure to bacterial infection and risk of schizophrenia. Schizophr Bull. 2009;35(3):631–7. 18832344 10.1093/schbul/sbn121 PMC2669577

[96] Jiang HY, et al. Maternal infection during pregnancy and risk of autism spectrum disorders: A systematic review and meta-analysis. Brain Behav Immun. 2016;58:165–72. 27287966 10.1016/j.bbi.2016.06.005

[97] Croen LA, et al. Infection and fever in pregnancy and autism spectrum disorders: findings from the study to explore early development. Autism Res. 2019;12(10):1551–61. 31317667 10.1002/aur.2175 PMC7784630

[98] Holingue C, et al. Interaction between maternal immune activation and antibiotic use during pregnancy and child risk of autism spectrum disorder. Autism Res. 2020;13(12):2230–41. 33067915 10.1002/aur.2411 PMC7839062

[99] Lee YH, et al. Effects of prenatal bacterial infection on cognitive performance in early childhood. Paediatr Perinat Epidemiol. 2020;34(1):70–9. 31837043 10.1111/ppe.12603

[100] Myka L, Estes AKM. Maternal immune activation: implications for neuropsychiatric disorders. Science. 2016;353(6301):772–7. 27540164 10.1126/science.aag3194 PMC5650490

[101] Knuesel I, et al. Maternal immune activation and abnormal brain development across CNS disorders. Nat Reviews Neurol. 2014;10(11):643–60. 10.1038/nrneurol.2014.187 25311587

[102] Giovanoli S, Weber L, Meyer U. Single and combined effects of prenatal immune activation and peripubertal stress on parvalbumin and reelin expression in the hippocampal formation. Brain, Behavior, and Immunity, 2014;40:48–54. 10.1016/j.bbi.2014.04.005 24859043

[103] Zheng Wa et al. Establishment of a two-hit mouse model of environmental factor induced autism spectrum disorder. Heliyon, 2024;10(9). 10.1016/j.heliyon.2024.e30617 PMC11107098 38774072

[104] Parenti I, et al. Neurodevelopmental disorders: from genetics to functional pathways. Trends Neurosci. 2020;43(8):608–21. 32507511 10.1016/j.tins.2020.05.004

[105] Cheslack-Postava K, Brown AS. Prenatal infection and schizophrenia: A decade of further progress. Schizophr Res. 2022;247:7–15. 34016508 10.1016/j.schres.2021.05.014 PMC8595430

[106] Shuid AN et al. Association between viral infections and risk of autistic disorder: an overview. Int J Environ Res Public Health, 2021;18(6). 10.3390/ijerph18062817 PMC7999368 33802042

[107] Dickerson F, et al. Schizophrenia is associated with an aberrant immune response to Epstein-Barr virus. Schizophr Bull. 2019;45(5):1112–9. 30462333 10.1093/schbul/sby164 PMC6737467

[108] Vianna P, et al. Zika virus as a possible risk factor for autism spectrum disorder: neuroimmunological aspects. Neuroimmunomodulation. 2018;25(5–6):320–7. 30630174 10.1159/000495660

[109] Figueiredo CP, et al. SARS-CoV-2-associated cytokine storm during pregnancy as a possible risk factor for neuropsychiatric disorder development in post-pandemic infants. Neuropharmacology. 2021;201:108841. 34666076 10.1016/j.neuropharm.2021.108841 PMC8519783

[110] Ousseny Zerbo YQ, Cathleen Yoshida JK, Grether J, Van de Water LA, Croen. Maternal infection during pregnancy and autism spectrum disorders. J Autism Dev Disord. 2016;45(12):4015–25. 10.1007/s10803-013-2016-3 PMC4108569 24366406

[111] Lee BH, et al. Activation of P2X(7) receptor by ATP plays an important role in regulating inflammatory responses during acute viral infection. PLoS ONE. 2012;7(4):e35812. 22558229 10.1371/journal.pone.0035812 PMC3338466

[112] García-Villalba J, et al. Soluble P2X7 Receptor Is Elevated in the Plasma of COVID-19 Patients and Correlates With Disease Severity. Front Immunol. 2022;13:894470. 35663992 10.3389/fimmu.2022.894470 PMC9161710

[113] Lécuyer D, et al. The purinergic receptor P2X7 and the NLRP3 inflammasome are druggable host factors required for SARS-CoV-2 infection. Front Immunol. 2023;14:1270081. 37920468 10.3389/fimmu.2023.1270081 PMC10619763

[114] Rogers JP, et al. Psychiatric and neuropsychiatric presentations associated with severe coronavirus infections: a systematic review and meta-analysis with comparison to the COVID-19 pandemic. Lancet Psychiatry. 2020;7(7):611–27. 32437679 10.1016/S2215-0366(20)30203-0 PMC7234781

[115] Vultaggio-Poma V, et al. The shed P2X7 receptor is an index of adverse clinical outcome in COVID-19 patients. Front Immunol. 2023;14:1182454. 37215142 10.3389/fimmu.2023.1182454 PMC10196164

[116] Ye Q, Wang B, Mao J. The pathogenesis and treatment of the `cytokine storm’ in COVID-19. J Infect. 2020;80(6):607–13. 32283152 10.1016/j.jinf.2020.03.037 PMC7194613

[117] Moore KM, Suthar MS. Comprehensive analysis of COVID-19 during pregnancy. Biochem Biophys Res Commun. 2021;538:180–6. 33384142 10.1016/j.bbrc.2020.12.064 PMC7759124

[118] Chen H, et al. Clinical characteristics and intrauterine vertical transmission potential of COVID-19 infection in nine pregnant women: a retrospective review of medical records. Lancet. 2020;395(10226):809–15. 32151335 10.1016/S0140-6736(20)30360-3 PMC7159281

[119] Dong L, et al. Possible vertical transmission of SARS-CoV-2 from an infected mother to her newborn. JAMA. 2020;323(18):1846–8. 32215581 10.1001/jama.2020.4621 PMC7099527

[120] Pantelis C, et al. Neurological, neuropsychiatric and neurodevelopmental complications of COVID-19. Aust N Z J Psychiatry. 2021;55(8):750–62. 32998512 10.1177/0004867420961472 PMC8317235

[121] Shanes ED, et al. Placental pathology in COVID-19. Am J Clin Pathol. 2020;154(1):23–32. 32441303 10.1093/ajcp/aqaa089 PMC7279066

[122] Shuffrey LC, et al. Association of birth during the COVID-19 pandemic with neurodevelopmental status at 6 months in infants with and without in utero exposure to maternal SARS-CoV-2 infection. JAMA Pediatr. 2022;176(6):e215563. 34982107 10.1001/jamapediatrics.2021.5563 PMC8728661

[123] Ayed M, et al. Neurodevelopmental outcomes of infants born to mothers with SARS-CoV-2 infections during pregnancy: a National prospective study in Kuwait. BMC Pediatr. 2022;22(1):319. 35637442 10.1186/s12887-022-03359-2 PMC9149327

[124] Jackson R, et al. Antenatal and neonatal exposure to SARS-CoV-2 and children’s development: a systematic review and meta-analysis. Pediatr Res. 2024;96(1):40–50. 38114608 10.1038/s41390-023-02954-y PMC11257989

[125] Erdogan MA, et al. Prenatal SARS-CoV-2 Spike protein exposure induces Autism-Like neurobehavioral changes in male neonatal rats. J Neuroimmune Pharmacol. 2023;18(4):573–91. 37889404 10.1007/s11481-023-10089-4

[126] de Araújo TVB, et al. Association between microcephaly, Zika virus infection, and other risk factors in brazil: final report of a case-control study. Lancet Infect Dis. 2018;18(3):328–36. 29242091 10.1016/S1473-3099(17)30727-2 PMC7617036

[127] Lottini G, et al. Zika virus induces FOXG1 nuclear displacement and downregulation in human neural progenitors. Stem Cell Rep. 2022;17(7):1683–98. 10.1016/j.stemcr.2022.05.008 PMC9287670 35714598

[128] Krow-Lucal ER, et al. Association and birth prevalence of microcephaly attributable to Zika virus infection among infants in paraíba, brazil, in 2015-16: a case-control study. Lancet Child Adolesc Health. 2018;2(3):205–13. 30169255 10.1016/S2352-4642(18)30020-8

[129] Azevedo RSS, et al. In situ immune response and mechanisms of cell damage in central nervous system of fatal cases microcephaly by Zika virus. Sci Rep. 2018;8(1):1. 29311619 10.1038/s41598-017-17765-5 PMC5758755

[130] Sherer ML, et al. Zika virus infection of pregnant rats and associated neurological consequences in the offspring. PLoS ONE. 2019;14(6):e0218539. 31220154 10.1371/journal.pone.0218539 PMC6586346

[131] Shi Y, et al. Vertical transmission of the Zika virus causes neurological disorders in mouse offspring. Sci Rep. 2018;8(1):3541. 29476066 10.1038/s41598-018-21894-w PMC5824946

[132] Elgueta D, et al. Consequences of viral infection and cytokine production during pregnancy on brain development in offspring. Front Immunol. 2022;13:816619. 35464419 10.3389/fimmu.2022.816619 PMC9021386

[133] Ohki CMY, et al. Zika virus infection impairs synaptogenesis, induces neuroinflammation, and could be an environmental risk factor for autism spectrum disorder outcome. Biochim Biophys Acta Mol Basis Dis. 2024;1870(5):167097. 38408544 10.1016/j.bbadis.2024.167097

[134] Leite-Aguiar R, et al. ATP-P2X7 signaling mediates brain pathology while contributing to viral control in perinatal Zika virus infection. Brain Behav Immun. 2024;118:318–33. 38460804 10.1016/j.bbi.2024.02.035

[135] Komal A, Noreen M, El-Kott AF. TLR3 agonists: RGC100, ARNAX, and poly-IC: a comparative review. Immunol Res. 2021;69(4):312–22. 34145551 10.1007/s12026-021-09203-6 PMC8213534

[136] Goasdoué K, et al. Review: the blood-brain barrier; protecting the developing fetal brain. Placenta. 2017;54:111–6. 27939102 10.1016/j.placenta.2016.12.005

[137] Szabó D, et al. Maternal P2X7 receptor inhibition prevents autism-like phenotype in male mouse offspring through the NLRP3-IL-1β pathway. Brain Behav Immun. 2022;101:318–32. 35065198 10.1016/j.bbi.2022.01.015

[138] Horváth G, et al. P2X7 Receptors Drive Poly(I:C) Induced Autism-like Behavior in Mice. J Neurosci. 2019;39(13):2542–61. 30683682 10.1523/JNEUROSCI.1895-18.2019 PMC6435822

[139] Haddad FL, De Oliveira C, Schmid S. Investigating behavioral phenotypes related to autism spectrum disorder in a gene-environment interaction model of Cntnap2 deficiency and Poly I:C maternal immune activation. Front Neurosci. 2023;17:1160243. 36998729 10.3389/fnins.2023.1160243 PMC10043204

[140] Bitanihirwe BK, et al. Late prenatal immune activation in mice leads to behavioral and neurochemical abnormalities relevant to the negative symptoms of schizophrenia. Neuropsychopharmacology. 2010;35(12):2462–78. 20736993 10.1038/npp.2010.129 PMC3055332

[141] Munarriz-Cuezva E, Meana JJ. Poly (I:C)-induced maternal immune activation generates impairment of reversal learning performance in offspring. J Neurochem. 2025;169(1):e16212. 39183542 10.1111/jnc.16212 PMC11657921

[142] Mueller FS, et al. Behavioral, neuroanatomical, and molecular correlates of resilience and susceptibility to maternal immune activation. Mol Psychiatry. 2021;26(2):396–410. 33230204 10.1038/s41380-020-00952-8 PMC7850974

[143] Choi GB, et al. The maternal interleukin-17a pathway in mice promotes autism-like phenotypes in offspring. Science. 2016;351(6276):933–9. 26822608 10.1126/science.aad0314 PMC4782964

[144] Ben-Reuven L, Reiner O. Dynamics of cortical progenitors and production of subcerebral neurons are altered in embryos of a maternal inflammation model for autism. Mol Psychiatry. 2021;26(5):1535–50. 31740755 10.1038/s41380-019-0594-y

[145] Bergdolt L, Dunaevsky A. Brain changes in a maternal immune activation model of neurodevelopmental brain disorders. Prog Neurobiol. 2019;175:1–19. 30590095 10.1016/j.pneurobio.2018.12.002 PMC6413503

[146] Crum WR, et al. Evolution of structural abnormalities in the rat brain following in utero exposure to maternal immune activation: A longitudinal in vivo MRI study. Brain Behav Immun. 2017;63:50–9. 27940258 10.1016/j.bbi.2016.12.008 PMC5441572

[147] Rasile M, et al. Maternal immune activation leads to defective brain-blood vessels and intracerebral hemorrhages in male offspring. Embo J. 2022;41(23):e111192. 36314682 10.15252/embj.2022111192 PMC9713716

[148] Casquero-Veiga M et al. The Poly I:C maternal immune stimulation model shows unique patterns of brain metabolism, morphometry, and plasticity in female rats. Front Behav Neurosci, 2023;16:1022622. 10.3389/fnbeh.2022.1022622 10.3389/fnbeh.2022.1022622 PMC9888250 36733452

[149] Coiro P, et al. Impaired synaptic development in a maternal immune activation mouse model of neurodevelopmental disorders. Brain Behav Immun. 2015;50:249–58. 26218293 10.1016/j.bbi.2015.07.022 PMC4955953

[150] Giovanoli S, et al. Prenatal immune activation causes hippocampal synaptic deficits in the absence of overt microglia anomalies. Brain Behav Immun. 2016;55:25–38. 26408796 10.1016/j.bbi.2015.09.015

[151] Corradini I, et al. Maternal immune activation delays Excitatory-to-Inhibitory Gamma-Aminobutyric acid switch in offspring. Biol Psychiatry. 2018;83(8):680–91. 29146047 10.1016/j.biopsych.2017.09.030

[152] Patrich E, et al. Maternal immune activation produces neonatal excitability defects in offspring hippocampal neurons from pregnant rats treated with Poly I:C. Sci Rep. 2016;6(1):19106. 26742695 10.1038/srep19106 PMC4705483

[153] Lee YH, et al. Maternal bacterial infection during pregnancy and offspring risk of psychotic disorders: variation by severity of infection and offspring sex. Am J Psychiatry. 2020;177(1):66–75. 31581799 10.1176/appi.ajp.2019.18101206 PMC6939139

[154] Kirsten TB, Bernardi MM. Prenatal lipopolysaccharide induces hypothalamic dopaminergic hypoactivity and autistic-like behaviors: repetitive self-grooming and stereotypies. Behav Brain Res. 2017;331:25–9. 28526515 10.1016/j.bbr.2017.05.013

[155] Carbone E et al. Maternal immune activation induced by prenatal lipopolysaccharide exposure leads to Long-Lasting Autistic-like social, cognitive and immune alterations in male Wistar rats. Int J Mol Sci, 2023;24(4). 10.3390/ijms24043920 PMC9968168 36835329

[156] Pålsson-McDermott EM, O’Neill LA. Signal transduction by the lipopolysaccharide receptor, Toll-like receptor-4. Immunology. 2004;113(2):153–62. 15379975 10.1111/j.1365-2567.2004.01976.x PMC1782563

[157] McDermott S, et al. Urinary tract infections during pregnancy and mental retardation and developmental delay. Obstet Gynecol. 2000;96(1):113–9. 10862853 10.1016/s0029-7844(00)00823-1

[158] Getahun D, et al. The effect of neonatal Sepsis on risk of autism diagnosis. Am J Perinatol. 2023;40(8):858–66. 34225371 10.1055/s-0041-1731648

[159] Martínez-García JJ, et al. P2X7 receptor induces mitochondrial failure in monocytes and compromises NLRP3 inflammasome activation during sepsis. Nat Commun. 2019;10(1):2711. 31221993 10.1038/s41467-019-10626-x PMC6586640

[160] Fan Z, et al. P2X7 receptor: A receptor closely linked with sepsis-associated encephalopathy. Open Life Sci. 2024;19(1):20220775. 38585633 10.1515/biol-2022-0775 PMC10998679

[161] Fialho S, et al. Could P2X7 receptor be a potencial target in neonatal sepsis? Int Immunopharmacol. 2024;142:112969. 39241519 10.1016/j.intimp.2024.112969

[162] Wang H, et al. P2RX7 sensitizes Mac-1/ICAM-1-dependent leukocyte-endothelial adhesion and promotes neurovascular injury during septic encephalopathy. Cell Res. 2015;25(6):674–90. 25998681 10.1038/cr.2015.61 PMC4456628

[163] Ozkanlar S, et al. P2X7 receptor antagonist A-438079 alleviates oxidative stress of lung in LPS-induced septic rats. Purinergic Signalling. 2023;19(4):699–707. 36959434 10.1007/s11302-023-09936-z PMC10754811

[164] Larrouyet-Sarto ML, et al. P2X7 receptor deletion attenuates oxidative stress and liver damage in sepsis. Purinergic Signal. 2020;16(4):561–72. 33090332 10.1007/s11302-020-09746-7 PMC7855213

[165] Savio LEB, et al. P2X7 Receptor Signaling Contributes to Sepsis-Associated Brain Dysfunction. Mol Neurobiol. 2017;54(8):6459–70. 27730511 10.1007/s12035-016-0168-9

[166] Alves VS, et al. P2X7 receptor contributes to long-term neuroinflammation and cognitive impairment in sepsis-surviving mice. Front Pharmacol. 2023;14:1179723. 37153798 10.3389/fphar.2023.1179723 PMC10160626

[167] Csóka B, et al. Extracellular ATP protects against sepsis through macrophage P2X7 purinergic receptors by enhancing intracellular bacterial killing. Faseb J. 2015;29(9):3626–37. 26060214 10.1096/fj.15-272450 PMC4550379

[168] Greve AS, et al. P2X(1), P2X(4), and P2X(7) Receptor Knock Out Mice Expose Differential Outcome of Sepsis Induced by α-Haemolysin Producing Escherichia coli. Front Cell Infect Microbiol. 2017;7:113. 28428949 10.3389/fcimb.2017.00113 PMC5382212

[169] Arranz-Solís D, Mukhopadhyay D, Saeij JJP. Toxoplasma effectors that affect pregnancy outcome. Trends Parasitol. 2021;37(4):283–95. 33234405 10.1016/j.pt.2020.10.013 PMC7954850

[170] Cabral CM, et al. Neurons are the primary target cell for the Brain-Tropic intracellular parasite Toxoplasma gondii. PLoS Pathog. 2016;12(2):e1005447. 26895155 10.1371/journal.ppat.1005447 PMC4760770

[171] Tyebji S, Hannan AJ, Tonkin CJ. Pathogenic infection in male mice changes sperm small RNA profiles and transgenerationally alters offspring behavior. Cell Rep. 2020;31(4):107573. 32348768 10.1016/j.celrep.2020.107573

[172] Brown AS, et al. Maternal exposure to toxoplasmosis and risk of schizophrenia in adult offspring. Am J Psychiatry. 2005;162(4):767–73. 15800151 10.1176/appi.ajp.162.4.767

[173] Bottari NB, et al. Resveratrol-mediated reversal of changes in purinergic signaling and immune response induced by Toxoplasma gondii infection of neural progenitor cells. Purinergic Signal. 2019;15(1):77–84. 30535987 10.1007/s11302-018-9634-3 PMC6439107

[174] Moreira-Souza ACA, Coutinho-Silva R. The complexity of purinergic signaling during Toxoplasma infection. Curr Top Med Chem. 2021;21(3):205–12. 33319661 10.2174/1568026621999201211202533

[175] Hughes HK, et al. Immune dysfunction and autoimmunity as pathological mechanisms in autism spectrum disorders. Front Cell Neurosci. 2018;12:405. 30483058 10.3389/fncel.2018.00405 PMC6242891

[176] Palmeira P et al. IgG placental transfer in healthy and pathological pregnancies. Clin Dev Immunol, 2012;985646. 10.1155/2012/985646 PMC3251916 22235228

[177] Chen SW, et al. Maternal autoimmune diseases and the risk of autism spectrum disorders in offspring: A systematic review and meta-analysis. Behav Brain Res. 2016;296:61–9. 26327239 10.1016/j.bbr.2015.08.035

[178] Zhu Z, et al. Maternal systemic lupus erythematosus, rheumatoid arthritis, and risk for autism spectrum disorders in offspring: A Meta-analysis. J Autism Dev Disord. 2020;50(8):2852–9. 32034648 10.1007/s10803-020-04400-y

[179] Brimberg L, et al. Brain-reactive IgG correlates with autoimmunity in mothers of a child with an autism spectrum disorder. Mol Psychiatry. 2013;18(11):1171–7. 23958959 10.1038/mp.2013.101

[180] Piras IS, et al. Anti-brain antibodies are associated with more severe cognitive and behavioral profiles in Italian children with autism spectrum disorder. Behav Immun. 2014;38:91–9. Brain. 10.1016/j.bbi.2013.12.020 PMC4111628 24389156

[181] Kaplan ZB, et al. Maternal thyroid dysfunction during pregnancy as an etiologic factor in autism spectrum disorder: challenges and opportunities for research. Thyroid^®^. 2023;34(2):144–57. 10.1089/thy.2023.0391 PMC10884547 38149625

[182] Escobar GMd., Obregón MJ, Rey FE, editors. Maternal thyroid hormones early in pregnancy and fetal brain development. Volume 18. Best Practice & Research Clinical Endocrinology & Metabolism; 2004. pp. 225–48. 2. 10.1016/j.beem.2004.03.012 15157838

[183] Whiteley P, et al. Autoimmune encephalitis and autism spectrum disorder. Front Psychiatry. 2021;12:775017. 34975576 10.3389/fpsyt.2021.775017 PMC8718789

[184] Martínez-Cerdeño V, et al. Prenatal exposure to Autism-Specific maternal autoantibodies alters proliferation of cortical neural precursor cells, enlarges brain, and increases neuronal size in adult animals. Cereb Cortex. 2016;26(1):374–83. 25535268 10.1093/cercor/bhu291 PMC4677982

[185] Ariza J, et al. Maternal autoimmune antibodies alter the dendritic arbor and spine numbers in the infragranular layers of the cortex. PLoS ONE. 2017;12(8):e0183443. 28820892 10.1371/journal.pone.0183443 PMC5562324

[186] Camacho J, et al. Embryonic intraventricular exposure to autism-specific maternal autoantibodies produces alterations in autistic-like stereotypical behaviors in offspring mice. Behav Brain Res. 2014;266:46–51. 24613242 10.1016/j.bbr.2014.02.045 PMC4075424

[187] Jha S, et al. The inflammasome sensor, NLRP3, regulates CNS inflammation and demyelination via caspase-1 and interleukin-18. J Neurosci. 2010;30(47):15811–20. 21106820 10.1523/JNEUROSCI.4088-10.2010 PMC6633756

[188] Grassi F, Salina G. The P2X7 receptor in autoimmunity. Int J Mol Sci, 2023;24(18). 10.3390/ijms241814116 PMC10531565 37762419

[189] Keystone EC, et al. Clinical evaluation of the efficacy of the P2X7 purinergic receptor antagonist AZD9056 on the signs and symptoms of rheumatoid arthritis in patients with active disease despite treatment with methotrexate or sulphasalazine. Ann Rheum Dis. 2012;71(10):1630–5. 22966146 10.1136/annrheumdis-2011-143578

[190] Eser A, et al. Safety and Efficacy of an Oral Inhibitor of the Purinergic Receptor P2X7 in Adult Patients with Moderately to Severely Active Crohn’s Disease: A Randomized Placebo-controlled, Double-blind, Phase IIa Study. Inflamm Bowel Dis. 2015;21(10):2247–53. 26197451 10.1097/MIB.0000000000000514

[191] Fiebich BL, Akter S, Akundi RS. The two-hit hypothesis for neuroinflammation: role of exogenous ATP in modulating inflammation in the brain. Front Cell Neurosci. 2014;8:260. 25225473 10.3389/fncel.2014.00260 PMC4150257

[192] Girard S, et al. IL-1 receptor antagonist protects against placental and neurodevelopmental defects induced by maternal inflammation. J Immunol. 2010;184(7):3997–4005. 20181892 10.4049/jimmunol.0903349

[193] Su Y, et al. Prenatal Poly I:C challenge affects behaviors and neurotransmission via elevated neuroinflammation responses in female juvenile rats. Int J Neuropsychopharmacol. 2022;25(2):160–71. 34893855 10.1093/ijnp/pyab087 PMC8832231

[194] Talukdar PM, et al. A proof-of-concept study of maternal immune activation mediated induction of Toll-like receptor (TLR) and inflammasome pathways leading to neuroprogressive changes and schizophrenia-like behaviours in offspring. Eur Neuropsychopharmacol. 2021;52:48–61. 34261013 10.1016/j.euroneuro.2021.06.009

[195] Szabo A, et al. Elevated levels of peripheral and central nervous system immune markers reflect innate immune dysregulation in autism spectrum disorder. Psychiatry Res. 2024;342:116245. 39481220 10.1016/j.psychres.2024.116245

[196] Suzuki K, et al. Plasma cytokine profiles in subjects with high-functioning autism spectrum disorders. PLoS ONE. 2011;6(5):e20470. 21647375 10.1371/journal.pone.0020470 PMC3103577

[197] O’Shea TM, et al. Elevated concentrations of inflammation-related proteins in postnatal blood predict severe developmental delay at 2 years of age in extremely preterm infants. J Pediatr. 2012;160(3):395–e4014. 22000304 10.1016/j.jpeds.2011.08.069 PMC3279610

[198] Bartha AI, et al. Neonatal encephalopathy: association of cytokines with MR spectroscopy and outcome. Pediatr Res. 2004;56(6):960–6. 15496611 10.1203/01.PDR.0000144819.45689.BB

[199] Bajnok A, et al. Distinct cytokine patterns May regulate the severity of neonatal asphyxia—an observational study. J Neuroinflamm. 2017;14(1):244. 10.1186/s12974-017-1023-2 PMC5727967 29233180

[200] Kelly SB, et al. Interleukin-1: an important target for perinatal neuroprotection? Neural Regen Res. 2023;18(1):47–50. 35799507 10.4103/1673-5374.341044 PMC9241389

[201] Stokes L, Spencer SJ, Jenkins TA. Understanding the role of P2X7 in affective disorders-are glial cells the major players? Front Cell Neurosci. 2015;9:258. 26217184 10.3389/fncel.2015.00258 PMC4495333

[202] Stokes L, et al. Two haplotypes of the P2X(7) receptor containing the Ala-348 to Thr polymorphism exhibit a gain-of-function effect and enhanced interleukin-1beta secretion. Faseb J. 2010;24(8):2916–27. 20360457 10.1096/fj.09-150862

[203] Backlund L, et al. P2RX7: expression responds to sleep deprivation and associates with rapid cycling in bipolar disorder type 1. PLoS ONE. 2012;7(8):e43057. 22952630 10.1371/journal.pone.0043057 PMC3429455

[204] Czamara D, Müller-Myhsok B, Lucae S. The P2RX7 polymorphism rs2230912 is associated with depression: A meta-analysis. Prog Neuropsychopharmacol Biol Psychiatry. 2018;82:272–7. 29122639 10.1016/j.pnpbp.2017.11.003

[205] Vereczkei A, et al. Association of purinergic receptor P2RX7 gene polymorphisms with depression symptoms. Prog Neuropsychopharmacol Biol Psychiatry. 2019;92:207–16. 30664971 10.1016/j.pnpbp.2019.01.006

[206] Wingo TS, et al. Brain proteome-wide association study implicates novel proteins in depression pathogenesis. Nat Neurosci. 2021;24(6):810–7. 33846625 10.1038/s41593-021-00832-6 PMC8530461

[207] Gonda X, et al. Significance of risk polymorphisms for depression depends on stress exposure. Sci Rep. 2018;8(1):3946. 29500446 10.1038/s41598-018-22221-z PMC5834495

[208] Soronen P, et al. P2RX7 gene is associated consistently with mood disorders and predicts clinical outcome in three clinical cohorts. Am J Med Genet B Neuropsychiatr Genet. 2011;156b(4):435–47. 21438144 10.1002/ajmg.b.31179

[209] Hansen T, et al. Variation in the purinergic P2RX7 receptor gene and schizophrenia. Schizophr Res. 2008;104(1):146–52. 18614336 10.1016/j.schres.2008.05.026

[210] Ripke S, et al. Biological insights from 108 schizophrenia-associated genetic loci. Nature. 2014;511(7510):421–7. 25056061 10.1038/nature13595 PMC4112379

[211] Feng S, et al. Mechanisms of NLRP3 activation and Inhibition elucidated by functional analysis of disease-associated variants. Nat Immunol. 2025;26(3):511–23. 39930093 10.1038/s41590-025-02088-9 PMC11876074

[212] Bhattacharya A, Jones DNC. Emerging role of the P2X7-NLRP3-IL1β pathway in mood disorders. Psychoneuroendocrinology. 2018;98:95–100. 30121550 10.1016/j.psyneuen.2018.08.015

[213] Grygorowicz T, Wełniak-Kamińska M, Strużyńska L. Early P2X7R-related astrogliosis in autoimmune encephalomyelitis. Mol Cell Neurosci. 2016;74:1–9. 26921791 10.1016/j.mcn.2016.02.003

[214] Sharp AJ, et al. P2x7 deficiency suppresses development of experimental autoimmune encephalomyelitis. J Neuroinflammation. 2008;5:33. 18691411 10.1186/1742-2094-5-33 PMC2518548

[215] Diaz-Hernandez JI, et al. In vivo P2X7 inhibition reduces amyloid plaques in Alzheimer’s disease through GSK3β and secretases. Neurobiol Aging. 2012;33(8):1816–28. 22048123 10.1016/j.neurobiolaging.2011.09.040

[216] Di Lauro C, et al. P2X7 receptor blockade reduces tau induced toxicity, therapeutic implications in tauopathies. Prog Neurobiol. 2022;208:102173. 34516970 10.1016/j.pneurobio.2021.102173

[217] Alves M, et al. P2X7R antagonism suppresses long-lasting brain hyperexcitability following traumatic brain injury in mice. Theranostics. 2025;15(4):1399–419. 39886340 10.7150/thno.97254 PMC11780721

[218] Garré JM, et al. P2X7 receptor inhibition ameliorates dendritic spine pathology and social behavioral deficits in Rett syndrome mice. Nat Commun. 2020;11(1):1784. 32286307 10.1038/s41467-020-15590-5 PMC7156443

[219] Bhattacharya A, et al. Pharmacological characterization of a novel centrally permeable P2X7 receptor antagonist: JNJ-47965567. Br J Pharmacol. 2013;170(3):624–40. 23889535 10.1111/bph.12314 PMC3792000

[220] Maenner MJ, et al. Prevalence and characteristics of autism spectrum disorder among children aged 8 Years - autism and developmental disabilities monitoring network, 11 sites, united states, 2020. MMWR Surveill Summ. 2023;72(2):1–14. 10.15585/mmwr.ss7202a1 PMC10042614 36952288

[221] Network GB. o.D.C. Global Burden of Disease Study 2019 (GBD 2019) Results. 2022 2025.05.28.]; Available from: https://vizhub.healthdata.org/gbd-results/

